# The PLK4 inhibitor RP-1664 demonstrates potent single-agent efficacy in neuroblastoma models through a dual mechanism of sensitivity

**DOI:** 10.21203/rs.3.rs-7014295/v1

**Published:** 2025-07-29

**Authors:** John Maris, Isabel Soria-Bretones, Matias Casás-Selves, Minu Samanta, David Groff, Jayne Murray, Jamie Fletcher, Alvin Farrel, Steven Pastor, Khushbu Patel, Elliot Goodfellow Goodfellow, Li Li, Cathy Caron, Ariya Shiwram, Hyeyeon Kim, Danielle Henry, Nancy Laterreur, Julian Bowlan, Kateryna Krytska, Steven Neuhauser, Timothy Stearns, Jeffrey Schubert, Jinhua Wu, Lea Surrey, Alejandro Álvarez-Quilón, Frédéric Vallée, Parham Nejad, Joseph Schonhoft, Joanna Li, Artur Veloso, Jordan Young, Marc Hyer, Stephen Morris, Yael (P.) Mossé, Gary Marshall, Michelle Haber, Michal Zimmermann

**Affiliations:** University of Pennsylvania and Children’s Hospital of Philadelphia; Repare Therapeutics, Inc., St. Laurent, QC, Canada; Repare Therapeutics, Inc., St. Laurent, QC, Canada; Children’s Hosp of Philadelphia; Children’s Hosp of Philadelphia; Children’s Cancer Institute; Children’s Cancer Institute Australia; Children’s Hospital of Philadelphia; Children’s Hospital of Philadelphia; Children’s Hosptial of Philadelphia; Repare Therapeutics, Inc., St. Laurent, QC, Canada; Repare Therapeutics, Inc., St. Laurent, QC, Canada; Repare Therapeutics, Inc., St. Laurent, QC, Canada; Repare Therapeutics, Inc., St. Laurent, QC, Canada; Repare Therapeutics, Inc., St. Laurent, QC, Canada; Repare Therapeutics, Inc., St. Laurent, QC, Canada; Repare Therapeutics, Inc., St. Laurent, QC, Canada; Repare Therapeutics, Inc., Cambridge, MA, USA; Children’s Hosptial of Philadelphia; The Jackson Laboratory; The Jackson Laboratory, Bar Harbor, ME, USA; Children’s Hospital of Philadelphia; Children’s Hospital of Philadelphia; Children’s Hospital of Philadelphia; Repare Therapeutics, Inc., St. Laurent, QC, Canada; Repare Therapeutics, Inc., St. Laurent, QC, Canada; Repare Therapeutics, Inc., Cambridge, MA, USA; Repare Therapeutics, Inc., Cambridge, MA, USA; Repare Therapeutics, Inc., St. Laurent, QC, Canada; Repare Therapeutics; Repare Therapeutics, Inc., St. Laurent, QC, Canada; Repare Therapeutics, Inc., Cambridge, MA, USA; Repare Therapeutics, Inc., St. Laurent, QC, Canada; Children’s Hospital of Philadelphia; Repare Therapeutics; Experimental Therapeutics Program; Repare Therapeutics

## Abstract

It was recently shown that inhibition of polo-like kinase 4 (PLK4) induces *TP53*-dependent synthetic lethality in cancers with chromosome 17q-encoded *TRIM37* copy number gain due to cooperative regulation of centriole duplication and mitotic spindle nucleation. We show here that chromosome 17q/TRIM37 gain is a pathognomonic feature of high-risk neuroblastoma and renders patient-derived cell lines hypersensitive to the novel PLK4 inhibitor RP-1664. We demonstrate that centriole amplification at low doses of RP-1664 contributes to this sensitivity in a *TRIM37*- and *TP53*-independent fashion. CRISPR screens and live cell imaging reveal that upon centriole amplification, neuroblastoma cells succumb to multipolar mitoses due to an inability to cluster or inactivate supernumerary centrosomes. RP-1664 showed robust anti-tumor activity in 14/15 neuroblastoma xenograft models and significantly extended survival in a transgenic murine neuroblastoma model. These data support biomarker-directed clinical development of PLK4 inhibitors for high-risk neuroblastoma and other cancers with somatically acquired *TRIM37* overexpression.

## INTRODUCTION

High-risk neuroblastoma is a childhood cancer with a 5-year survival rate of approximately 50% despite dose-intensive chemotherapy including autologous stem cell transplantations, surgery, radiation therapy, and immunotherapy.^1^ Genomic gain of the long arm of chromosome 17 (17q) by unbalanced translocation is among the most recurrent molecular alterations in neuroblastoma^2–6^, but the exact prevalence of this genomic aberration as well as its biologic and clinical relevance remains poorly defined. Chromosome 17q gain often spans a region between 17q11 and 17q25, which contains the *TRIM37* gene located at 17q22–23^2,7,8^. Recently, *TRIM37* genomic gain and high expression was shown to confer a cancer therapeutic vulnerability due to synthetic lethality with inhibition of polo-like kinase 4 (PLK4)^9,10^.

PLK4 is a dimeric serine-threonine kinase that plays key roles in centrosome formation^11^. The centrosome, the main microtubule-organizing center of the cell, is composed of two centrioles and the peri-centriolar material (PCM), which together nucleate microtubules that form the mitotic spindle^11,12^. The formation of centrosomes is tightly regulated. Each mitotic cell normally has two centrosomes, which drive bipolar division and equal separation of genetic material (as well as centrosomes) into two daughter cells. In turn, each G1 daughter cell contains one centrosome with one centriole doublet that is duplicated in S-phase, ensuring that the subsequent mitosis will again contain two centrosomes^12^. PLK4 is critical for centriole duplication: it interacts with, and phosphorylates, several centriolar and PCM proteins to drive *de novo* procentriole assembly on the parent centriole^13–17^. Consequently, PLK4 downregulation leads to progressive loss of centrioles, whereas PLK4 overexpression leads to centriole amplification and supernumerary centrosomes^13,14,18^. To guarantee that centriole duplication occurs only once per cell cycle, PLK4 also regulates its own stability: trans-autophosphorylation of several serine and threonine residues within a PLK4 dimer activates degradation of the protein by the SCF-bTrCP ubiquitin ligase^18–21^. Catalytically inactive PLK4 therefore accumulates in the cell^18–21^.

Synthetic lethality between high TRIM37 levels and PLK4 inhibition is based on their complementary roles in centrosome biogenesis^9,10^. PLK4 inhibition causes centriole loss, for which normal cells compensate in part by using the PCM to nucleate the mitotic spindle^9,10^. However, as *TRIM37* encodes an E3-ubiquitin ligase that negatively regulates the stability of multiple PCM components through proteasomal degradation^9,10,22^, *TRIM37* overexpression compromises the PCM’s integrity. PLK4 inhibition in the context of high TRIM37 abundance thus leads to depletion of both centrioles and the PCM, an inability to form the mitotic spindle, and mitotic failure or delay^9,10^. Prolonged mitosis is sensed by the mitotic surveillance (or ‘stopwatch’) pathway driven by the USP28–53BP1-p53 axis, which triggers a p21-mediated cell cycle arrest and cell death^9,10,23–26^. Based on this synthetic lethality paradigm, we developed RP-1664: a selective, potent, and orally bioavailable PLK4 inhibitor^27^, which is presently being evaluated in a first-in-human Phase 1 clinical trial for patients with refractory solid malignancies showing gain of the *TRIM37* locus (NCT06232408).

The effect of pharmacologic PLK4 inhibition on centriole biogenesis is bimodal. Whereas complete PLK4 inactivation at higher concentrations of PLK4i leads to centriole loss, lower concentrations induce centriole amplification^28–31^. This centriole amplification has been attributed to an intermediate inhibition state of PLK4 with enough inactive PLK4 monomers existing to reduce trans-autophosphorylation and increase total PLK4 levels, but not enough PLK4 molecules are inhibited to suppress centriole duplication^28,32,33^.

Here we show that centriole overduplication driven by low concentrations of RP-1664 contribute to PLK4i-sensitivity of neuroblastoma tumor cells, independent of *TRIM37* and *TP53*. This is due to the fact that at low doses of RP-1664 neuroblastoma cells are unable to compensate for supernumerary centrosomes, whereas at higher doses there is the anticipated centriolar loss and mitotic delay. We then show that 17q gain including the *TRIM37* locus is a near universal feature of high-risk neuroblastoma and that RP-1664 shows robust single agent efficacy across a panel of human neuroblastoma cell line derived xenograft (CDX) and patient-derived xenograft (PDX) models, as well as a genetically engineered mouse model (GEMM) of this disease, supporting clinical development of PLK4 inhibition strategies for high-risk neuroblastoma.

## RESULTS

### RP-1664 is a PLK4 inhibitor with pre-clinical activity against TRIM37-amplified and TP53 wild-type tumors.

**Figure 1:**
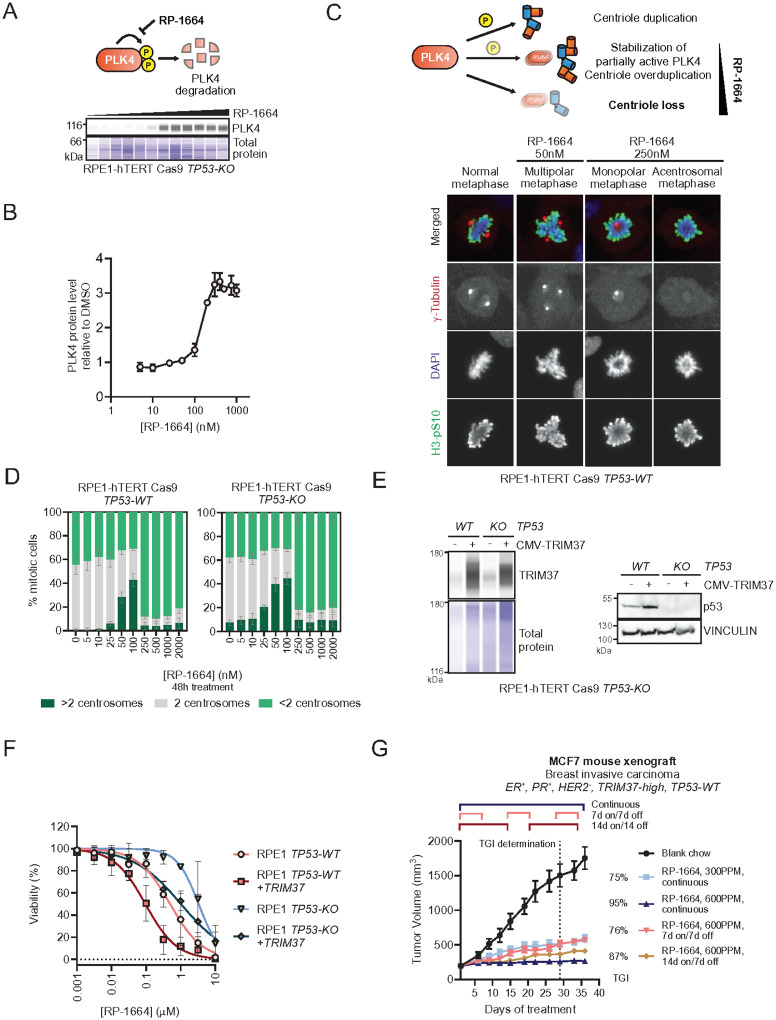
RP-1664 is a potent and selective PLK4i. A,B. RP-1664 induces PLK4 stabilization. A. *Top*: Model of PLK4 self-regulation. PLK4 autophosphorylation leads to its degradation. This is blocked by RP-1664. *Bottom*: Representative capillary immunodetection of PLK4 in RPE1-hTERT Cas9 *TP53*-*KO* whole cell extracts. Total protein shown as a loading control. B. Representative (of *N*=3 independent experiments) quantification of PLK4 protein levels in capillary immunodetection assays. Mean of two technical replicates from one representative experiment ±SD. C,D. RP-1664 modulates centrosome biogenesis. C. *Top*: Schematic of bimodal modulation of centriole numbers by RP-1664. Low concentrations induce centriole amplification, higher concentrations lead to centriole loss. *Bottom*:Representative micrographs of RPE1-hTERT Cas9 *TP53*-*KO* cells after no treatment or treatment with indicated RP-1664 concentrations and immunofluorescence staining with γ-Tubulin (visualizing centrosomes) and H3-pS10 (mitotic marker) antibodies. DAPI is a nuclear counterstain. D. Quantification of mitotic RPE1 Cas9 *TP53*-*WT* and *KO* cells with <2, 2, and >2 centrosomes at indicated RP-1664 concentrations in *N*=3 independent experiments. Mean value (bars) is shown ±SD. E-G. WT p53 and high TRIM37 sensitize to RP-1664. E. Representative (of N≥2 independent experiments) TRIM37 capillary immunodetection (left) and *TP53* immunoblot (right) of RPE1-hTERT Cas9 *TP53*-*WT* and KO cells with or without CMV-TRIM37 overexpression. Total protein and vinculin are loading controls. F. Dose-response of RP-1664 on growth (measured by Incucyte) of RPE1 *TP53*-*WT* and *KO* cells, with or without CMV-TRIM37. Mean of *N*=3 independent experiments ±SD. Solid lines show a non-linear regression fit to a four-parameter dose-response model. G. Tumor volume measurements of MCF7 mouse xenograft tumors in animals fed blank chow or RP-1664-containing chow at indicated doses and schedules. TGI = percent tumor growth inhibition relative to blank chow. Mean of *N*=6 mice/group ±SEM.

RP-1664 is a novel, selective and orally bioavailable PLK4 inhibitor^27^. To confirm that RP-1664 inhibits PLK4 in human cells, we assessed its ability to induce PLK4 stabilization, modulate centrosome numbers, and activate the *TP53* mitotic surveillance pathway (Figure 1A–D; **Extended Data Figure 1A,B**).

First, we analyzed the effect of RP-1664 on PLK4 protein level, as inhibition of PLK4 counteracts its SCF-bTrCP-mediated degradation^18–21^. As expected, RP-1664 treatment led to a dose-dependent increase in total PLK4 in RPE1-hTERT Cas9 *TP53*-null cells^34^ when measured by capillary-based immunodetection, with a maximal effect observed above 100 nM and partial PLK4 stabilization occurring below this concentration (Figure 1A,B). To determine whether RP-1664 disrupts PLK4-dependent centriole biogenesis, we quantified the number of centrosomes (visualized by anti-γ-Tubulin immunofluorescence^35^) per mitosis (marked by phosphorylated serine 10 on histone H3 – H3pS10^36^) using high-content fluorescence microscopy (Figure 1C,D). In both *TP53*-proficient and -deficient RPE1 cells, RP-1664 induced centrosome loss at concentrations >100 nM, with the majority of mitotic cells showing one or no centrosomes after ~2 population doublings (Figure 1C,D). Concentrations between ~25–100nM induced supernumerary centrosomes, as expected from partial PLK4 inhibition^28^ (Figure 1C,D). Finally, to explore whether RP-1664 activates the mitotic surveillance pathway, we stained *TP53*-wild type (WT) RPE1 cells for the p53 transcriptional target CDKN1A/p21 (**Extended Data Figure 1A**). RP-1664 increased p21 expression in a dose-dependent manner, at concentrations in agreement with those inducing PLK4 stabilization and modulating centrosome numbers (**Extended Data Figure 1A,B**). In aggregate, we show that RP-1664 induces cellular phenotypes expected from a PLK4i^28,29,31,37^.

To evaluate the contributions of *TRIM37* and *TP53* to RP-1664 sensitivity^9,10^, we overexpressed the TRIM37 open reading frame in RPE1-hTERT Cas9 *TP53*-*WT* and *TP53*-*KO* cells. Lentiviral TRIM37 transduction led to a 3–5x increase in TRIM37 protein level (Figure 1E; **Extended Data Figure 1C**). We then compared the RP-1664 sensitivity of TRIM37-overexpressing cells to parental *TP53*-*WT* and *TP53*-*KO* cells by real-time monitored growth assays (Incucyte), revealing a ~30-fold increase in sensitivity from *TRIM37*-normal/*TP53*-*KO* cells to TRIM37-high/*TP53*-*WT* cells, with TRIM37-high/*TP53*-*KO* and TRIM37-normal/*TP53*-*WT* cells showing intermediate sensitivity (Figure 1F). Concentrations of RP-1664 that completely inhibited growth of TRIM37-high/*TP53*-*WT* cells were above 100nM, consistent with the doses of RP-1664 that induce centrosome loss (Figure 1D). These data confirm that high TRIM37 level and functional p53 cooperatively increase cellular sensitivity to PLK4 inhibition by RP-1664.

To determine the activity of RP-1664 against *TRIM37*-*high TP53*-WT tumors *in vivo* we engrafted MCF7 breast carcinoma cells subcutaneously into immunodeficient mice. MCF7 cells express estrogen and progesterone receptors but no human epithelial growth factor receptor (ER^+^, PR^+^, HER2^−^), carry a *TRIM37*-gain, are *TP53*-*WT*, and have been previously shown to be sensitive to PLK4i in a *TRIM37*-dependent manner^9^. For successful implantation, mice were irradiated and supplemented with estradiol in their drinking water. Mice with implanted tumors were treated with RP-1664 delivered in chow at multiple doses and schedules (Figure 1G), leading to dose-dependent anti-tumor activity of RP-1664 with maximal tumor growth inhibition (TGI) of 95% at 600 parts per million (ppm) RP-1664 chow (Figure 1G). RP-1664 demonstrated schedule flexibility, with an intermittent 14 days on/7days off schedule showing only modest reduction in efficacy compared to continuous delivery (Figure 1G). All doses and schedules were well tolerated with only one animal showing body weight (BW) loss of >20% (22% on the last day of dosing) in the continuous 600 ppm cohort, despite estradiol supplementation negatively impacting body weight loss in all groups (**Extended Data Figure 1D**). We conclude that RP-1664 shows efficacy in a pre-clinical tumor model with TRIM37 gain and WT *TP53*.

### Chromosome 17q gain is a pathognomonic biomarker of high-risk neuroblastoma.

**Figure 2.**
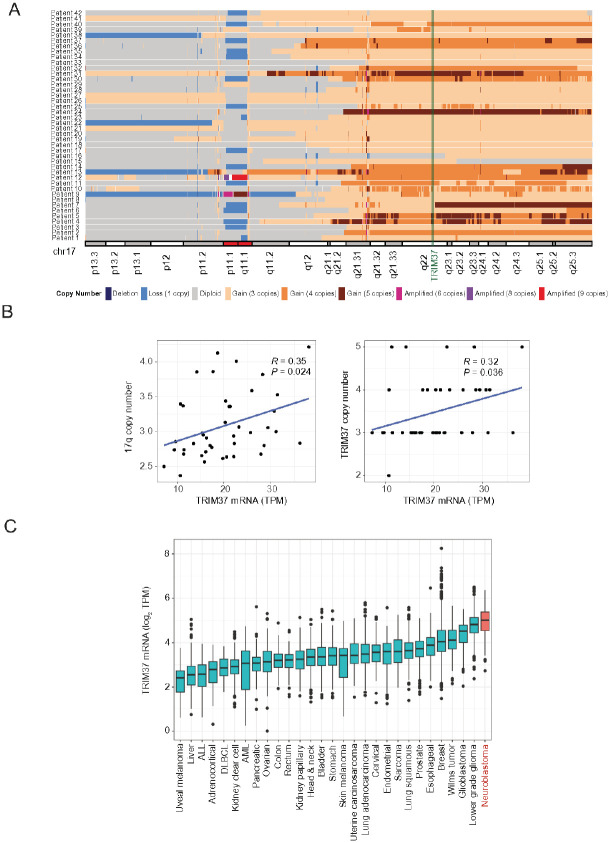
*TRIM37* gain is a pathognomonic feature of high-risk neuroblastoma. A. Copy number variation across chromosome 17 in 42 high-risk neuroblastomas from the Gabriella Miller Kids First (GMKF) patient cohort. Colors indicate copy number as described below the plot. Cytogenetic map of chromosome 17 is shown for reference. B. Correlation between *TRIM37* mRNA expression and 17q (left) or *TRIM37* (right) copy number in the GMFK neuroblastoma dataset as in A. P values were calculated using a T-test based on the Pearson’s correlation coefficient *(R)* and the sample size *(N)* where the $T\text{-statistic}=R\cdot \sqrt{\left(N-2\right)/\left(1-R^{2}\right)}$. C. *TRIM37* mRNA expression across tumor indications in the Open Targets dataset.

The reported high prevalence of 17q gain in neuroblastoma presents a compelling rationale for development of a PLK4i in this disease, but estimates of the exact frequency of this aberration vary widely in the literature^2–6^, perhaps due to evolving genomic analytic technologies. To define the frequency of *TRIM37* copy number gain in high-risk neuroblastoma we analyzed whole genome sequencing data from the Gabriella Miller Kids First (GMFK) cohort of primary diagnostic neuroblastomas (dbGaP Study Accession: phs001436.v1.p1). After extensive quality control to remove cases with low tumor content and/or low sequencing coverage (see Methods), all 42 high-risk neuroblastomas showed evidence for 17q gain (37 with segmental 17q gain, 5 with whole chromosome 17 gain), with all but one sample showing gain at the *TRIM37* locus (Figure 2A). To further validate this finding, we reviewed 45 consecutive newly diagnosed high-risk neuroblastoma samples submitted for NGS using the CHOP solid tumor gene panel^38^. All 45 cases showed 17q gain including the TRIM37 locus by inference of surrounding genes on the NGS panel (39 with segmental 17q gain, 6 with whole chromosome 17 gain). Finally, *TRIM37* copy number and mRNA levels were directly associated and neuroblastoma showed the highest median mRNA expression level of all human cancers (Figure 2B,C).

### *TRIM37/TP53*-dependent and -independent PLK4i sensitivity in neuroblastoma cells

**Figure 3:**
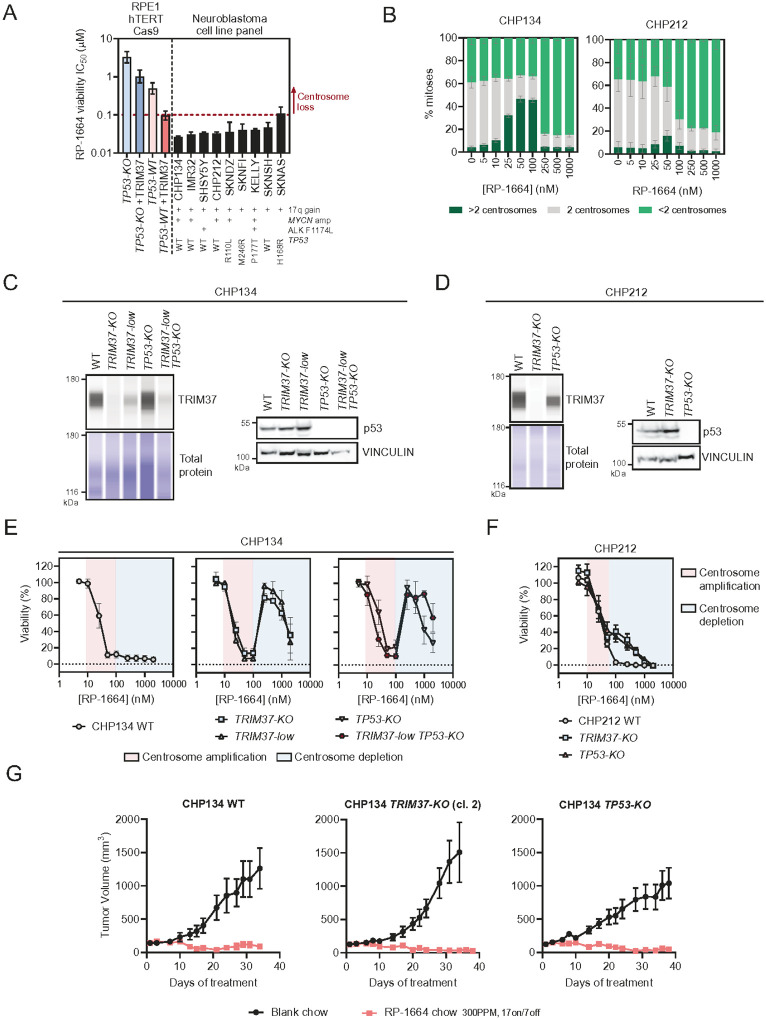
TRIM37- and p53-independent sensitivity of neuroblastoma cells to centrosome amplification. A. RP-1664 cell growth IC_50_ values for indicated cell lines. Data from non-linear least square fitting of mean viability values (N≥3 independent experiments) in growth (Incucyte (RPE1, CHP134) or CellTiter Glo (all others)) assays ±95% confidence interval. Dashed line shows the lowest concentration inducing centrosome loss in RPE1 cells. B. Quantification of mitotic CHP134 and CHP212 cells with <2, 2, and >2 centrosomes at indicated RP-1664 concentrations in N=3 independent experiments. Mean value (bars) is shown ±SD. C,D. Representative TRIM37 capillary immunodetection (left) and p53 immunoblots (right) of CHP134 (C) and CHP212 (D) of indicated genotypes. Total protein and vinculin are loading controls. E,F. Cell viability of CHP134 (E; measured by Incucyte) and CHP212 (F; CellTiter Glo) cells of indicated genotypes treated with indicated RP-1664 concentrations. Pink area shows concentrations causing centrosome amplification, blue represents centrosome depletion. Mean of N=3 to 5 independent experiments ±SD. G. Tumor volume measurement of CHP134 WT, *TRIM37*-*KO* and *TP53*-*KO* mouse xenografts treated with blank chow or 300ppm RP-1664 chow using a 17d on / 7d off schedule. Mean of 7 mice/group ±SEM.

Due to near universal prevalence of *TRIM37* gain in neuroblastoma, we sought to define the potency of RP-1664 in preclinical models of this disease. We observed high sensitivity to RP-1664 across a panel of nine neuroblastoma cell line models carrying *TRIM37* gain, with a median viability IC_50_ of 35nM (range 26–109nM; Figure 3A). *TP53* mutated cell lines tended to show a higher IC_50_, but the trend was subtle (median for *TP53* WT vs. *TP53* mutant: 33 and 40nM, respectively; *P* = 0.11, Mann-Whitney test). Interestingly, the median IC_50_ across all cell lines was below the 100nM concentration that is required for depletion of centrosomes in RPE1 cells and instead correlated with centrosome amplification (Figure 3A,B). This was not due to cell type-dependent differences in modulation of centrosome number by RP-1664, as we observed centrosome amplification between 10 to ~50–100nM, and centrosome loss at or above 100nM RP-1664 not only in RPE1 cells, but also in three different neuroblastoma cell lines (Figure 3B; **Extended Data Figure 2A,B**). Since it is centriole loss that underlies the sensitivity of TRIM37-high and WT p53 cells to PLK4i^9,10^, we hypothesized that cytotoxicity observed in neuroblastoma cells at low RP-1664 concentrations is independent of TRIM37 and p53 status. To test this hypothesis, we generated *TRIM37*- and *TP53*-CRISPR/Cas9 *KO* clones in the neuroblastoma cell lines CHP134 and CHP212 (Figure 3C,D) – as well as a clone of CHP134, referred to as *TRIM37*-*low*, that retains reduced TRIM37 expression due to residual wild-type alleles (Figure 3C; **Extended Data Figure 2C**). We also made a CHP134 *TRIM37*-*low*/*TP53*-*KO* cell line, which has p53 inactivated on top of lowered TRIM37 levels (Figure 3C). We then compared RP-1664 sensitivity of these cell lines to parental WT cells (Figure 3E,F).

Inactivation of p53, lowering TRIM37 levels, and/or ablating TRIM37 completely, reduced RP-1664 sensitivity of both CHP134 and CHP212 cells at concentrations associated with centrosome loss, but were unable to induce resistance to RP-1664 at lower doses that lead to centrosome amplification (Figure 3E,F). The rescue of sensitivity to higher, but not lower, PLK4i doses by reducing TRIM37 level in CHP134 cells was reproduced with the published selective PLK4i Centrinone B^37^, ruling out compound-specific effects (**Extended Data Figure 2D-F**). Cellular sensitivity to different RP-1664 concentrations correlated with p53 activation: whereas RP-1664 induced p21 expression in CHP134 cells at doses leading to centrosome amplification as well as depletion, lowering TRIM37 levels dampened p21 expression only at centrosome depletion concentrations (**Extended Data Figure 3A,B**). Furthermore, whereas a dose of RP-1664 causing centrosome depletion prolonged mitotic duration in CHP134 cells but not CHP134 *TRIM37*-*low* as expected from the TRIM37-PLK4 synthetic lethality model^10,23^, a dose causing centrosome amplification did not (**Extended Data Figure 3C,D**), again suggesting a different mechanism of action. Sensitivity of CHP134 and CHP212 cells to different RP-1664 concentrations was also associated with induction of apoptosis and cell death as measured by Annexin V and Cytotox (reflective of membrane permeability) staining (**Extended Data Figure 3E,F**). Altogether, our data suggest that neuroblastoma cells are sensitive to lower and higher PLK4i concentrations in two distinct ways: at lower doses, neuroblastoma cells are killed by PLK4i regardless of *TRIM37* and *TP53* status, whereas at higher doses neuroblastoma sensitivity depends on high TRIM37 and functional p53.

### *TRIM37*- and *TP53*-independent sensitivity of neuroblastoma tumors to RP-1664 in vivo.

To test whether the *TRIM37*- and *TP53*-independent mechanism of sensitivity contributes to anti-tumor activity of RP-1664 *in vivo*, we engrafted immunodeficient mice with parental CHP134 cells, alongside a *TRIM37*-*KO* clone (different than the one used in our *in vitro* studies but showing the same RP-1664 sensitivity - **Extended Data Figure 4A**) and a *TP53*-*KO* clone. We confirmed that the resulting tumors maintained the desired TRIM37 and p53 status (**Extended Data Figure 4B**) and treated tumor-bearing mice with either blank chow or 300ppm RP-1664 chow, which in this model led to a daily free plasma RP-1664 concentration of ≤100nM on average, consistent with centriole amplification (**Extended Data Figure 4C**). This regimen led to robust efficacy in the parental CHP134 model, with regression of 6/7 tumors (Figure 3G). Interestingly, inactivation of neither *TRIM37* nor *TP53* affected the efficacy of RP-1664 (Figure 3G), suggesting that the TRIM37 and p53-independent mechanism of sensitivity in CHP134 cells is active *in vivo*. The treatment was well tolerated in all models with no weight loss observed (**Extended Data Figure 4D**). These data suggests that low doses of RP-1664 are sufficient to elicit *TRIM37*- and *TP53*-independent anti-tumor activity *in vivo*.

### CRISPR screens confirm a dual mechanism of RP-1664 sensitivity

**Figure 4:**
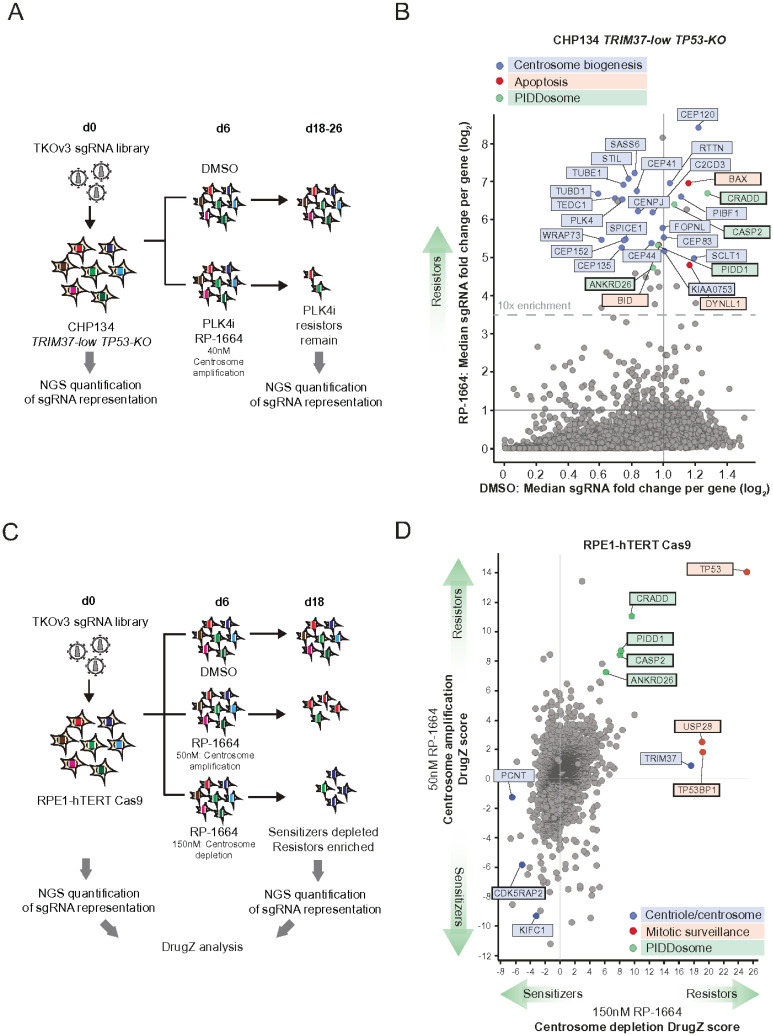
CRISPR screens for genes modulating sensitivity to RP-1664. A,B. Screen for gene knockouts causing RP-1664 resistance in CHP134 *TRIM37*-*low*/*TP53*-*KO* cells. A. Experimental design. See methods for details. B. Screen results. Median sgRNA fold changes per gene in RP-1664-treated cells (Y axis) vs. untreated (X axis). Resistor hits with known functions in centriole/centrosome biogenesis (blue), apoptosis (red) or the PIDDosome (green) are highlighted. C,D. Screen for genes knockouts causing RP-1664 resistance or sensitivity in RPE1-hTERT Cas9 cells. C. Experimental design. See methods for details. D. Screen results. Gene-level DrugZ^85^ scores in cells treated with 50nM RP-1664 (centrosome amplification; Y axis) vs. 150nM (centrosome depletion; X axis). Hits with known functions in centriole/centrosome biogenesis (blue), the mitotic surveillance pathway (red) or the PIDDosome (green) are highlighted.

Our cellular studies implied that neuroblastoma cells are sensitive to low concentrations of PLK4i that lead to centrosome amplification. We therefore asked whether it is centrosome amplification *per se* that underlies neuroblastoma sensitivity. To that end we utilized unbiased CRISPR/Cas9-enabled screening, to map TRIM37- and p53-independent mechanisms of neuroblastoma sensitivity to RP-1664. We transduced CHP134 *TRIM37*-*low*/*TP53*-*KO* cells with the genome-wide TKOv3 Cas9/sgRNA library^39,40^, and treated the resulting cell pool with 40nM RP-1664 (dose leading to centrosome amplification) or DMSO as a control. Over the course of the screen 40nM RP-1664 resulted in ~80% loss of cell number as compared to DMSO-treated cells. We used NGS to determine which sgRNAs were enriched in the final RP-1664-treated cell population over the initial cell pool, as these are likely to cause resistance to the compound (Figure 4A, Methods). We identified several genes with median sgRNA representation increased upon RP-1664 compared to DMSO treatment (Figure 4B, **Supplementary Table 1**). Analyzing known functions of these genes revealed striking patterns: Out of 41 genes showing at least 10-fold median sgRNA enrichment, 20 are involved in centrosome biology, 3 regulate apoptosis, and 4 are components of the PIDDosome complex (Figure 4B). The presence of multiple centrosomal components (e.g. *STIL, CEP120, CEP152* etc.), as well as *PLK4* itself, in the list of hits suggests that blunting the cells’ ability to produce centrosomes alleviates RP-1664 cytotoxicity. The PIDDosome triggers apoptosis upon recognising supernumerary centrosomes^41,42^ and therefore its inactivation likely allows CHP134 cells to survive despite centrosome amplification. The PIDDosome component caspase 2 is known to induce apoptosis through the pro-apoptotic BCL2 family of factors including BID and BAX^43^, both of which scored as hits in our screen (Figure 4B). In aggregate, these data implicate centrosome amplification as the *bona fide* cause of TRIM37- and p53-independent cell death in CHP134 neuroblastoma cells at low concentrations of RP-1664.

To further explore the genetic framework of response to RP-1664, we performed a chemogenomic screen in Cas9-expressing RPE1-hTERT *TP53*-*WT* cells treated with DMSO, 50nM RP-1664 (centrosome amplification), or 150nM RP-1664 (centrosome depletion) (Figure 4C,D; **Supplementary Table 2**). The lower sensitivity of RPE1 cells to RP-1664 compared to CHP134 allowed us to determine not only which gene knockouts lead to RP-1664 resistance, but also which genes, when inactivated, increase RP-1664 sensitivity. The results were well in agreement with a published screen performed using Centrinone B^28^ (**Supplementary Table 2**). We successfully identified *TRIM37*, and the mitotic surveillance factors *USP28* and *TP53BP1*, as required for cell sensitivity to the dose of RP-1664 leading to centrosome depletion. At the same time, PIDDosome components were among the strongest resistor hits at the lower concentration (Figure 4D; note that the PIDDosome factors scored as hits also in the higher dose arm, albeit not as strongly as *TRIM37* or *TP53BP1*). These data support the interpretation that cells can become sensitive to RP-1664 by two orthogonal mechanisms - centrosome amplification and depletion.

Of note, in RPE1 cells, inactivation of *TP53* caused resistance to both doses of RP-1664 (Figure 4D). This was confirmed by a screen using cytosine-to-adenine base editor (CBE^FNLS^)^44^-expressing RPE1 cells, and an adaptation of a published sgRNA library that generates 5855 single-nucleotide variants in 298 cancer-related genes (Extended Data Figure 5A).^45^ *TP53* mutations caused resistance to both 50nM and 150nM RP-1664 (**Extended Data Figure 5B,C; Supplementary Table 3**). It should be noted, however, that RPE1 cells with normal TRIM37 levels show only minimal sensitivity to 50 nM RP-1664 (see Figure 1F) suggesting that enrichment of cells carrying *TP53* sgRNAs in our CRISPR screens may be reflective of altered growth kinetics of *TP53*-deficient cells upon low-dose PLK4i as compared to *TP53*-proficient ones, rather than a bona fide rescue of cell death.

### Neuroblastoma cells lack mechanisms to tolerate excess centrosomes

Among the genes whose inactivation sensitized to 50nM RP-1664 in RPE1 cells was *KIFC1* (Figure 4D). Its gene product, KIFC1 (or HSET) is a kinesin motor protein previously implicated in cellular tolerance to supernumerary centrosomes^46,47^. KIFC1 facilitates centrosome clustering if more than two centrosomes are present, enabling formation of a (pseudo-)bipolar spindle^47^. The inability to cluster supernumerary centrosomes may thus underlie sensitivity to low PLK4i concentrations.

**Figure 5:**
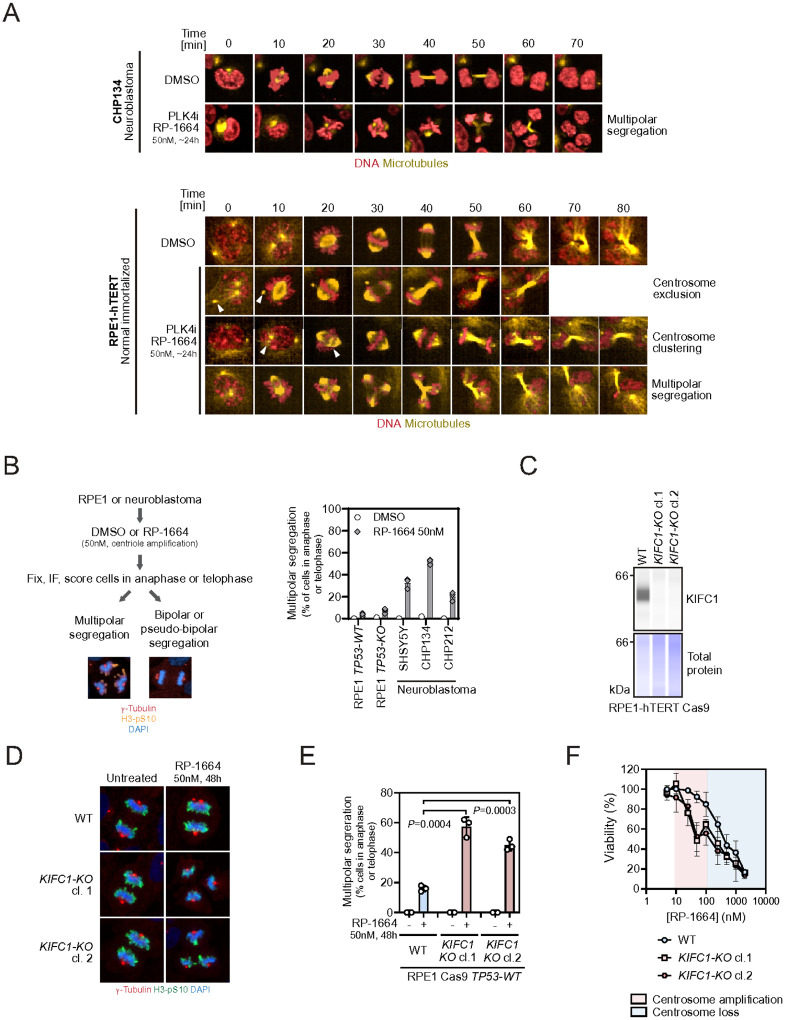
Lack of clustering/exclusion of extra centrosomes leads to RP-1664 sensitivity. A. Representative tempograms from time-lapse imaging of CHP134 (top) and RPE1-hTERT Cas9 (bottom) cells stained with SPY555-Tubulin for microtubules (yellow) and SPY650-DNA for DNA (red) and treated with DMSO or 50nM RP-1664. Example cells undergoing normal division, multipolar segregation, or pseudo-bipolar division with centrosome clustering or centrosome exclusion are shown. B. Left: Workflow for quantification of multipolar segregation frequency. RPE1 or neuroblastoma cells were treated with 50nM RP-1664, fixed and immunostained for centrosomes (g-Tubulin) and mitosis (H3-pS10). DAPI was a nuclear counterstain. Anaphase and telophase cells undergoing either bipolar or multipolar division were quantified. Right: Frequency of multipolar segregation in anaphase or telophase with or without RP-1664 in indicated cell lines. Data from *N*=3 independent experiments (open symbols) with mean (bars) ±SD. C. Representative KIFC1 capillary immunodetection of *KIFC1*-*WT* and *KIFC1*-*KO* RPE1-hTERT Cas9 cells. Total protein is a loading control. D. Representative micrographs of *KIFC1*-*WT* and KO cells processed for immunofluorescence with g-Tubulin and H3-pS10 antibodies with or without RP-1664 treatment. DAPI used as a nuclear counterstain. E. Quantification of *KIFC1*-*WT* and *KO* cells in anaphase or telophase undergoing multipolar vs. bipolar division in presence or absence of RP-1664. Data from *N*=3 independent experiments (open symbols) with mean (bars) ±SD. *P* values determined with an unpaired two-tailed T-test. F. RP-1664 sensitivity of *KIFC1*-*WT* and *KIFC1*-*KO* cells. Mean viability measurements from *N*=3 independent Incucyte growth assays ±SD.

To test this hypothesis, we stained DNA and microtubules in CHP134 cells with fluorescent dyes (SPY650-DNA and SPY555-Tubulin) and followed mitotic progression with or without 50nM RP-1664 by live cell imaging. As a ‘normal’ cell control we used RPE1-hTERT *TP53*-*WT* cells. Whereas under unperturbed conditions CHP134 cells divided normally, we observed multipolar segregation once cells were treated with RP-1664 at low nM concentrations (Figure 5A; **Supplementary Video 1,2**). In contrast, RPE1-hTERT cells displayed a wider range of phenotypes upon RP-1664 treatment: In addition to occasional multipolar segregation, RPE1 cells also performed pseudo-bipolar mitoses with supernumerary centrosomes, utilizing either centrosome clustering or exclusion (Figure 5A; **Supplementary Video 3–5**) – both known mechanisms of adaptation to extra centrosomes^48–50^. To quantify the frequency of multipolar and bipolar mitoses in neuroblastoma versus normal cells upon RP-1664 treatment, we fixed and stained with anti-γ-Tubulin and H3-pS10 antibodies three neuroblastoma cell lines (CHP134, CHP212, SHSY5Y), as well as RPE1 controls, and counted the frequency of cells in anaphase or telophase that underwent bipolar versus multipolar segregation (Figure 5B). Whereas immortalized RPE1 cells infrequently displayed multipolar mitosis in presence of 50nM RP-1664, multipolar mitoses were common in neuroblastoma cells, suggesting that it is indeed the inability to cope with supernumerary centrosomes that underlies PLK4i hypersensitivity (Figure 5B). To examine whether disrupting centrosome clustering would sensitize the normally resistant RPE1 cells to low dose PLK4i, we CRISPR-inactivated *KIFC1* in RPE1-hTERT Cas9 *TP53*-*KO* cells and confirmed that the frequency of multipolar cell divisions robustly increased in two *KIFC1*-*KO* clones upon 50nM RP-1664 treatment (Figure 5C–E). Consistent with our CRISPR screen data, *KIFC1*-*KO* cells were more sensitive to doses of RP-1664 causing centrosome amplification than parental cells, whereas sensitivity to doses leading to centrosome depletion was comparable (Figure 5F). We conclude that the ability to cluster (or inactivate) supernumerary centrosomes governs cellular sensitivity to partial PLK4 inhibition.

### RP-1664 shows potent single-agent efficacy in human neuroblastoma-derived xenograft models.

**Figure 6:**
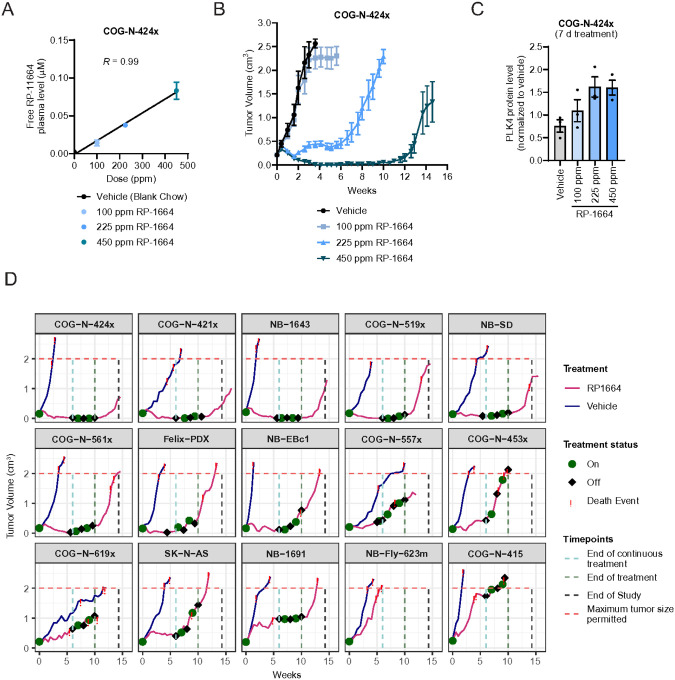
Potent single-agent efficacy of RP-1664 in neuroblastoma xenograft models. A. Free (not bound to plasma protein) plasma concentrations of RP-1664 in mice treated with indicated doses of RP-1664 chow. Mean of *N*=3 mice. B. Tumor volume measurement of COG-N-424x mouse xenografts treated with blank chow or indicated doses of RP-1664 chow using a xxx schedule continuous dosing schedule of RP-1664 for six weeks, followed by two cycles of one week on and one week off as detailed in method section. Mean of N=6 mice/group ±SEM. C. PLK4 protein level quantification by capillary immunodetection in lysates from COG-N-424x tumors treated for 7 days with indicated doses of RP-1664. Mean of *N*=3 mice ±SEM. D. Tumor volume measurements in 15 xenograft models of high-risk neuroblastoma treated with vehicle or 450 ppm of RP-1664 chow. Treatment periods and endpoint events are indicated. Data are mean of N=3 mice/group.

Next, we sought to determine whether the dual sensitivity of neuroblastoma cells to centrosome amplification and depletion translates broadly into anti-tumor activity and tolerability *in vivo*. We deployed a panel of fifteen human neuroblastoma xenograft models (5 CDX and 10 PDX), all showing 17q gain including the *TRIM37* locus (**Supplementary Table 4;** no xenograft models without 17q gain were available). To explore the range of efficacious RP-1664 doses, we first performed a dose-response study in one of our models, COG-N-424x, at 100, 225, and 450 ppm RP-1664 chow. The RP-1664 chow formulation showed pharmacokinetic dose-linearity across 100 ppm, 225 ppm, and 450 ppm (Figure 6A).

After three days of dosing, 225 and 450 ppm achieved a free plasma RP-1664 concentration of 38 ±5 and 83 ±19 nM, respectively (Figure 6A) – at or above the median cell line IC_50_ previously measured in growth assays. Consistent with this observation, RP-1664 showed a dose-dependent anti-tumor activity, with minimal tumor growth inhibition at 100ppm, tumor regression at 450ppm, and intermediate efficacy at 225ppm (Figure 6B, **Extended Data Figure 6A,B**). To assess whether this efficacy correlated with target engagement and modulation by RP-1664, we analyzed PLK4 protein stabilization and p21 expression in COG-N-424x tumors by capillary immunodetection and immunohistochemistry, respectively. Doses that induced anti-tumor activity elevated both PLK4 and p21 protein levels in a dose- and time-dependent manner, confirming target modulation by RP-1664 at biologically active exposures (Figure 6C and **Extended Data Figure 6C-E**). Target engagement was also confirmed in a second model, COG-N-421 (**Extended Data Figure 6F-H**).

Next, we explored RP-1664 efficacy across the entire fifteen-model panel. We chose a 450 ppm dose of RP-1664, which elicited tumor regression in our dose-response experiment and was in a historically well-tolerated range on both continuous and intermittent schedules (Figure 1G, Valée et al., 2025, J. Med. Chem. *in press*). Mice with established xenografts averaging 200mm^3^ were randomized to be exposed to RP-1664 chow at 450ppm or standard chow, N=3/arm, with the treated mice being exposed continuously for 42 days, followed by an intermittent, 7 days on/7 off, schedule until day 70. Using response criteria established by the Pediatric Preclinical Testing Consortium,^51,52^ 14/15 (93%) of the xenografts showed evidence for anti-tumor activity, with an objective response rate (ORR) of 53% (seven complete responses, two of which were maintained), one partial response, and six significant growth delays extending survival (Table 1, Figure 6D, **Extended Data Figure 7A,B**). RP-1664 was generally well tolerated, with mice harboring three xenografts requiring brief interruptions in dosing due to >15% weight loss (**Extended Data Figure 7C**). No clear biomarker of RP-1664 response was evident from review of the most obvious candidates and it is notable that two *TP53* mutated models (COG-N-519x, NB-SD) showed robust responses (Figure 6D).

### RP-1664 increases survival in an immunocompetent mouse model of neuroblastoma

**Figure 7:**
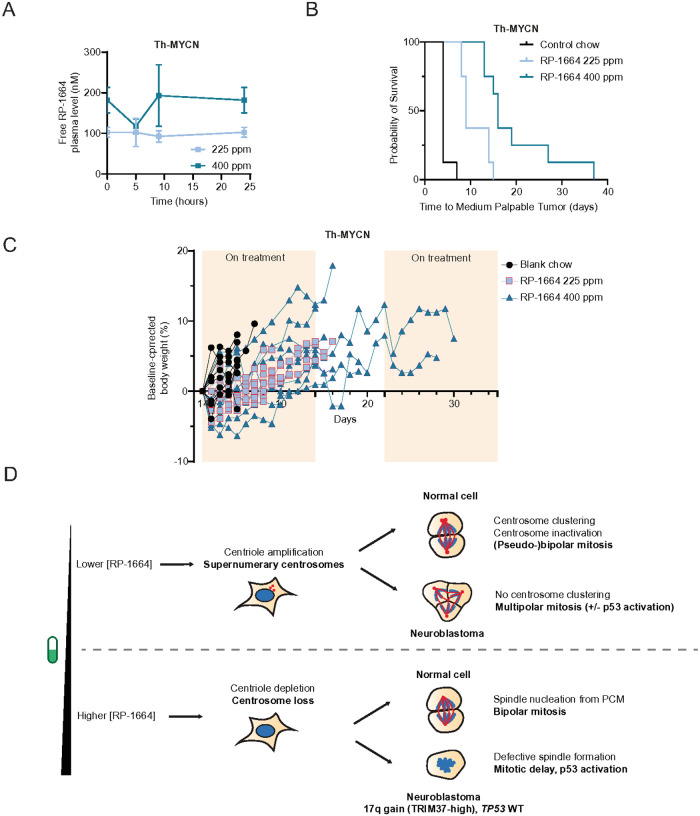
RP-1664 extends survival of immunocompetent mice with spontaneous neuroblastomas. A. Free (not bound to plasma protein) plasma concentrations of RP-1664 in Th-MYCN mice treated with indicated doses of RP-1664 chow. Mean of *N*=3 mice at indicated time points ±SEM. B. Kaplan-Meier survival curves showing survival of Th-MYCN mice treated with RP-1664 at presentation of small palpable tumor. *N*=8 mice/group. C. Percentage weight loss of mice treated with RP-1664 over time. Data show individual mice. D. A two-component model of neuroblastoma sensitivity to PLK4i. See main text for details.

Finally, we aimed to assess the efficacy of RP-1664 in a genetically engineered model of neuroblastoma. The Th-MYCN mouse model, targeting human *MYCN* expression to the neural crest, recapitulates poorly differentiated late-stage neuroblastoma in an immunocompetent mouse^53,54^. All mice homozygous for the *MYCN* transgene develop tumours by 7 weeks of age^54,55^. We treated tumor-bearing mice with control chow, or RP-1664 chow at 225 and 400ppm. As we observed sporadic body weight loss in our prior xenograft panel upon continuous dosing, we delivered the RP-1664 chow on an intermittent, 14 day on / 7 day off schedule. In the Th-MYCN model these doses led to average free RP-1664 plasma concentrations of 100±5nM and 169±34nM (Figure 7A), covering concentrations associated with centriole amplification and loss, respectively. Mice treated with RP-1664 demonstrated extension of survival in a dose-dependent manner. Mice on control chow had an average survival of 4.375 days which was extended to 10.75 and 19.5 days in the 225ppm and 400ppm RP-1664 treated groups, respectively (n=8/arm). Treatment significantly extended survival in both treatment groups over the control group (P<0.0001 in both cases) (Figure 7B). The higher dose also significantly extended survival over the lower dose (P=0.0031). Tumor regression was observed in 3/8 animals on the higher dose, while the remainder demonstrated slower tumor progression. Weight loss was minimal over the course of treatment; however, weights did tend to fluctuate when mice cycled on and off the medicated chow (Figure 7C). We conclude that tolerated doses of RP-1664 are broadly efficacious across multiple pre-clinical models of high-risk neuroblastoma.

## DISCUSSION

PLK4 inhibition was suggested as a therapeutic strategy for high-risk neuroblastoma due to a high prevalence of *TRIM37* gain^10^. We now demonstrate that 17q/*TRIM37* gain is more prevalent than previously reported, suggesting that selection of neuroblastoma patients based on *TRIM37* copy number status may not be necessary. We then showed that the PLK4i RP-1664 therapy results in robust single agent anti-tumor activity across a spectrum of high-risk neuroblastoma pre-clinical models reflecting the genetic heterogeneity of this disease. However, this exquisite PLK4 sensitivity cannot be attributed solely to *TRIM37* overexpression. We uncovered that PLK4i sensitivity of neuroblastoma cells is in fact a composite of two complementary but distinct mechanisms. At lower doses, PLK4i (including RP-1664) amplify centrosomes through stabilization of partially active PLK4^28–33^. Neuroblastoma cells are unable to tolerate supernumerary centrosomes, as they do not employ centrosome clustering or exclusion. In turn neuroblastoma cells undergo multipolar segregation, presumably leading to aneuploidy and genomic instability (Fig. 7D). On the other hand, at higher PLK4i concentrations neuroblastoma cells lose centrosomes and succumb to mitotic delay or failure as a consequence of high TRIM37 levels^10^ (Fig. 7D). This dual mechanism makes neuroblastoma an attractive target population for PLK4i like RP-1664 and adds to a growing list of factors predictive of PLK4i sensitivity, including high *TRIM37* levels^9,10^ or b-Catenin hyperactivation^56^, the latter of which is seldomly observed in high-risk neuroblastoma.

We demonstrate that centrosome clustering is a *bona fide* mechanism of resistance to PLK4i, as inactivation of a centrosome clustering factor, *KIFC1*, sensitizes normally resistant cells to low dose RP-1664. However, an important outstanding question is what exact genetic basis underlies the inability to compensate for supernumerary centrosomes in neuroblastoma. In addition to *KIFC1*, multiple genetic pathways have been previously described to facilitate centrosome clustering^48^, including factors involved in microtubule organization, the spindle assembly checkpoint, sister chromatid cohesion, as well as the actin cytoskeleton and cell adhesion^47,49,57–61^. However, it is unclear whether defects in any of these mechanisms contribute to neuroblastoma tumorigenesis. Similarly, it will be important to determine whether any of the recurrent genetic alterations in neuroblastoma such as *MYCN* amplification^1^, *ALK* gain-of-function mutation/amplification^1^, *LIN28B* overexpression^62,63^, and chromosome 1p or 11q loss^64^, contribute to defects in centrosome clustering. Multiple potential links between these alterations and centrosome biology exist. For example, we showed that *LIN28B* overexpression has been linked to overactivation of the mitotic kinase Aurora A (AURKA)^62^ involved in centrosome maturation, spindle assembly and cytokinesis^65^. *MYCN* amplification has been previously associated with centrosome amplification^66,67^, but another study did not corroborate these data^68^. Future correlative studies using our high-risk neuroblastoma models may prove useful in uncovering the genetic mechanisms that govern PLK4i sensitivity or resistance. Of note, in a recent pre-print, Moreno-Marin *et al*. describe a remarkable diversity in centrosome clustering proficiency among the NCI60 cancer cell line panel, suggesting that sensitivity to centrosome overduplication may be found in additional tumor lineages^69^.

Our study also brings further support for a complex role of the p53 pathway in the response to abnormal centrosome numbers. Whereas in neuroblastoma cells p53 was dispensable for cell death at doses of RP-1664 causing centrosome amplification, p53 inactivation reduced sensitivity of RPE1 cells at equivalent RP-1664 concentrations. The simplest reconciliation of this apparent discrepancy is that cell death upon centrosome amplification can occur both in a p53-dependent as well as independent fashion, consistent with the complete responses in two *TP53* mutant xenograft models. Our CRISPR screens implicate the PIDDosome in mediating RP-1664 cytotoxicity. Canonically, the PIDDosome is activated by recruitment to distal appendages of supernumerary centrioles by ANKRD26, leading to caspase 2-mediated cleavage of the p53 inhibitor MDM2^70^. However, caspase 2 can trigger apoptosis also in a p53-independent manner, through release of pro-apoptotic factors (such as BID or BAX) from mitochondria.^71^ It is therefore possible that this pathway contributes to PLK4i cytotoxicity in neuroblastoma when p53 is inactivated, which is consistent with our CRISPR screen observations.

The single agent anti-tumor activity seen in this study with an ORR of 53% compares favorably with the historical ORR of 14% seen in the NCI preclinical testing program^72^. Indeed, across the 20-year history of this program for *in vivo* screening of anti-cancer agents, the majority of drug showed an ORR of 0, with higher response rates in more recent years to antibody drug conjugates with potent payloads^51^. The most robust single agent activity reported to date in high-risk neuroblastoma preclinical models is the ALK inhibitor lorlatinib specific to ALK mutated models^73^, where a clear mechanistic basis was tested and translated to robust clinical activity^74^. With the extremely high prevalence of 17q gain in high-risk neuroblastoma, there is a tremendous opportunity for biomarker directed clinical development of RP-1664 with ongoing and future emphasis on optimal combination strategies and defining mechanisms of acquired resistance.

## Methods

### Cell culture

Cell lines were purchased from the following vendors: RPE1, MCF7, CHP212, SHSY5Y, SKNAS, SKNDZ, SKNFI, SKNSH - ATCC; KELLY – Sigma Aldrich; CHP134, IMR32 – DSMZ. RPE1-hTERT Cas9 *TP53*-*WT* and *TP53*-*KO* were described before^34,75^. Cells were cultured at 37°C and 5% CO_2_ in the following media (all supplemented with 10% fetal bovine serum (VWR, 76419–584), 100 U/ml penicillin and 100 mg/ml streptomycin (Corning, 30–001-CI)): RPE1, CHP212, CHP134, SKNDZ, SKNFI – DMEM (Corning, 10–013-CV); IMR32, SKNAS, KELLY – RPMI (Corning, 10–104-CV); MCF7 – EMEM (Corning, 10–009-CV) + 1% non-essential amino acids + 10mg/ml insulin; SKNSH – MEM (Corning 10–010-CV); SHSY5Y – MEM : F12K (1:1; Corning 10–090-CV).

### Genomic and transcriptomic data analysis

The whole genome sequencing data (WGS) utilized in this project was obtained through the Gabriella Miller Kids First Project (dbGaP phs001436.v1.p1)^76^. Genomic sequencing was performed at Hudson Alpha Institute for Biotechnology (DNA) and St. Jude Children’s Research Hospital’s Genomic Sequencing Laboratory (RNA). WGS was performed using Illumina’s HiSeq X System at a mean coverage of 30x. Whole exome sequencing (WES) was performed using Illumina’s HiSeq 4000 System at a mean coverage of 150x. Total RNA sequencing (RNA-seq) was performed using Illumina’s HiSeq 4000 System.

Tumor DNA samples with a confirmed sequencing coverage of at least 20x were subjected to consensus somatic copy number variant (CNV) analysis. Tumor/normal sample pair concordance was verified using NGSCheckMate^77^. CNV calling was performed using Control-FREEC^78^, CNVkit^79^, and GATK^80^ to enable consensus-based CNV detection, as previously described^81^. RNA-Seq data comprising a minimum of 20 million total reads, with at least 50% of reads successfully aligning to the human genome. RNA reads were aligned to the HG38 reference genome using STAR^82^. Gene level quantification was performed using RSEM^83^ with gene annotations based on Gencode release v39.

Validation of 17q copy number gain status in CHOP tumor samples was undertaken under Children’s Hospital of Philadelphia Institutional Review Board (IRB) protocol 25–023583. Sequencing was performed using targeted capture DNA sequencing using Illumina’s HiSeq 4000 at a mean coverage of 2000x. Sequence data was analyzed for copy number analysis using NextGENe V2 NGS analysis software and visual inspection.

### CRISPR/Cas9 knockout of *TRIM37, TP53, KIFC1*

Cells were transfected with Cas9:sgRNA complexes using Lipofectamine CRISPRmax (Thermo Fisher Scientific) according to manufacturer’s protocol, allowed to recover for 2–3 days and then seeded for clonogenic outgrowth. Knockout (KO) clones were characterized by Sanger sequencing, ICE analysis^84^ and/or immunoblotting/Simple Western analysis. The following sgRNA target sequences were used: *TRIM37* – TCGCATCAGTGTGCACTTTG or GATGAAGTAAATCAGCTCGA; *TP53* – CAGAATGCAAGAAGCCCAGA; *KIFC1* – GTCCCCCCTATTGGAAGTAA.

### Chemical compounds

RP-1664 was synthesized in house (synthesis will be described in a separate manuscript). Centrinone B was purchased from MedChem Express. Compounds were made up at stock concentrations of 10 mM from powder in dimethyl sulfoxide (DMSO) and kept at −20°C for long-term storage.

### Simple Western capillary immunodetection

For cell lysate preparation, 150,000 cells/well were plated in 12-well plates (Falcon, 353043). Where applicable, increasing doses of RP-1664 were added to the cells using a Tecan D300E dispenser. 48 hours later, cells were washed with 1 ml PBS and lysed in-plate with 50 μl of RIPA buffer (Sigma, R0278–50ML), supplemented with 1X protease and phosphatase inhibitors (Thermo-Fisher, 78440).

Xenograft tumor lysates were prepared as follows: Lysis buffer (MSD Tris + 1x protease and phosphatase inhibitors) was added to tumor samples at a w/v ratio of 1:9 (tumor : buffer) and tissue was homogenized using an OMNI bead ruptor 24 bioruptor (speed 5.5 m/s, 3 cycles, 10 s/cycle, 3 min break between cycles on ice). Homogenate was transferred to fresh Eppendorf tubes and cleared by centrifugation at 13,000RPM, 5 min at 4°C. Clarification steps were repeated until lysate was completely clear.

Following lysis, protein concentrations were measured using a DC Protein Assay Kit (Bio-Rad, 5000112) and protein levels in all samples were normalized to 1 μg/μl. Immunodetection was performed using a Simple Western JESS instrument (Bio-Techne) according to the manufacturer’s instructions. 3 μl of lysate was loaded per capillary and proteins were detected with their respective primary antibodies (see below). Separation time was set to 25 min, voltage to 375V, primary antibody incubation to 90 min, and secondary antibody incubation to 60 min.

### Immunoblotting

Cells were lysed in 2x Novex Tris-Glycine SDS sample buffer + 200mM DTT (Thermo Fisher Scientific LC2676) at 1×10^6^ cells/ml and boiled at 95°C for 5 min. 20–30ml of lysate were run on Novex Tris-Glycine SDS gels (Thermo Fisher Scientific). Proteins were transferred to 0.2mM nitrocellulose membranes at 90V for 2–2.5h. Membranes were blocked with 5% milk/TBST for 30 min at room temperature (RT) and incubated with primary antibodies at 4°C overnight. Membranes were washed 3× 5min with TBST and incubated with secondary antibodies for 1h at RT, after which they were washed and developed using the Pierce ECL Pico or Femto Western Blotting substrate (Thermo Fisher Scientific). Chemiluminescence was detected on an Amersham Imager 680 instrument (GE).

### Immunofluorescence

Cells were seeded on black, clear bottom, poly-D-lysine coated 96-well plates (PhenoPlate 96; Revvity, 6055500) and RP-1664 was added the following day using a Tecan D300E dispenser. 48–72 h later, cells were rinsed with PBS and fixed with 4% paraformaldehyde/PBS (PFA; Thermo Fisher, J19943.K2) for 10 mins at RT. Plates were rinsed 2x with PBS and stored at 4°C. For immunofluorescent staining, cells were permeabilized with 0.3% Triton X-100/PBS for 30 min, and rinsed 2x with PBS. Plates were blocked with blocking buffer (5% goat serum / 0.1% Triton X-100 / PBS) for 1 h. Cells were then incubated overnight at 4°C or 2h at RT with primary antibodies in blocking buffer. Next day, plates were rinsed 3x with 0.1% Triton X-100/PBS and incubated for 1h at RT with secondary antibodies supplemented with 0.5 mg/ml 4′,6-diamidino-2-phenylindole (DAPI). Plates were rinsed 3x with 0.1% Triton X-100/PBS and fresh PBS was added to image on an Operetta CLS or Opera Phenix automated high-content microscope in confocal mode, using a 40x NA1.1 water immersion objective (Revvity).

### Cell growth assays

Cells were seeded on white, clear bottom 96-well plates (Corning, 3903) and compounds were added the next day using a Tecan D300E dispenser. The following seeding densities were used (cells/well): RPE1 – 300; CHP134 – 1500; CHP212 – 1000; SHSY5Y – 1000; IMR32 – 700; SKNFI – 3000; SKNSH – 500; SKNDZ – 2000; KELLY – 700; SKNAS – 700–800. When cells reached near-confluence (~4–6 population doublings / ~6–12 days), plates were imaged on an Incucyte S3 automated microscope. Images were analyzed using the Incucyte 2022B software to determine % confluence. Alternatively, cells were processed using the CellTiter Glo cell viability assay (Promega) according to manufacturer’s instructions. Luminescence was measured with a FlexStation 3 plate reader. Data were normalized to untreated controls and IC_50_ values were obtained by non-linear least squares fitting of the data to a four-parameter dose-response model in GraphPad PRISM 10.

### Cytotox and Annexin V cell viability assays

Cells were seeded on black poly-lysine coated 96-well plates (Revvity, 6055500). Two days later, cells were incubated with Incucyte dyes to detect cell death (Cytotox; Sartorius 4633; 1:500) or apoptosis (Annexin V; Sartorius 4642; 1:2000). RP-1664 was added to the plate using Tecan D300E. Plates were imaged on an Incucyte S3 instrument in both phase-contrast and green fluorescent channels every 8 hours for 5 days and analyzed using Incucyte 2022B software. Cell death induction by RP-1664 was calculated as the % area stained with green dye (Cytotox or Annexin V) normalized to % confluence, and relative to DMSO.

### Antibodies

The following antibodies and dilutions were used for immunoblotting (IB), immunofluorescence (IF) or capillary immunodetection (JESS): rabbit anti-PLK4 E6A7R (Cell Signaling Technologies 71033; JESS 1:200), rabbit anti-TRIM37 (Bethyl A301–174A; JESS 1:50), mouse anti-p53 DO-1 (sc-126; IB 1:1000), rabbit anti-p21 12D1 (Cell Signaling Technologies 2947; IF 1:500, JESS 1:300), rabbit anti-g-Tubulin EPR16793 (Abcam 179503; IF 1:500), mouse anti-H3pS10 3H10 (Sigma Aldrich 05–806; IF 1:1000), rabbit anti-KIFC1 (ProteinTech 20790–1-AP; JESS 1:50), rabbit anti-DYKDDDDK tag (FLAG) D6W5B (Cell Signaling Technologies 14793; IB 1:1000), rabbit anti-Vinculin E1E9V (Cell Signaling Technologies 13901; IB 1:5000), Alexa Fluor 488/555/647-conjugated goat anti-rabbit or anti-mouse IgG (H+L) (Thermo Fisher Scientific A-11008/A-21428/A-21245/A-11001/A-21422/A-21235; IF 1:500–1:1000), HRP-conjugated goat anti-mouse IgG (BioRad L005680; 1:5000 IB), HRP-conjugated goat anti-rabbit IgG (Jackson ImmunoResearch 111–035-144; IB 1:5000), HRP-conjugated anti-rabbit secondary antibody (Bio-Techne 042–206; JESS undiluted).

### Live cell imaging

Cells were seeded on 96-well poly-lysine coated PhenoPlates (Revvity, 6055500) and incubated in phenol red-free media supplemented with SPY650-DNA (Cytoskeleton CY-SC501) and SPY555-Tubulin (Cytoskeleton CY-SC203) at 1:6000 dilution for 2 hours before imaging. RP-1664 or DMSO treatments were added 24 hours prior and maintained during imaging. Time-lapse images were captured on an Opera Phenix automated high-content microscope in confocal mode at 37°C and 5% CO_2_, using a 40x NA1.1 water immersion objective (Revvity). Images were captured every 4–5 minutes for ~6 hours and analyzed manually using Harmony. Mitotic duration was recorded for each dividing cell, counted as the time from nuclear envelope breakdown to mitotic exit (i.e., anaphase or chromatin decondensation).

### CRISPR/Cas9 chemogenomic screening

CRISPR/Cas9 screens were performed as previously described^40^. The RP-1664 resistance screen was performed in a CHP134 *TRIM37*-*low TP53*-*KO* neuroblastoma cell line. Cells were transduced with lentivirus carrying the TKOv3 sgRNA library at a multiplicity-of-infection (MOI) of ~0.3. The screen was conducted in technical duplicates, and library coverage of >100 cells per sgRNA was maintained at every step. Puromycin-containing medium (0.5 μg/ml) was added 2 days after infection to select for transductants. Selection was continued until 96 h after infection, which was considered the initial time point (*t*_0_). 40nM RP-1664 was added to the cells starting from day 6 (*t*_6_). From *t*_10_ onwards, RP-1664-containing medium was refreshed every four days until the screen was terminated at *t*_26_. To identify genes whose deletion caused resistance to RP-1664, genomic DNA was isolated from surviving cells using the QIAamp Blood Maxi Kit (Qiagen) and genome-integrated sgRNA sequences were amplified by PCR using NEBNext Ultra II Q5 Master Mix (New England Biolabs). i5 and i7 multiplexing barcodes were added in a second round of PCR and final gel-purified products were sequenced on an Illumina NextSeq500 system to determine sgRNA representation in each sample.

The RPE1-hTERT Cas9 *TP53*-*WT* screen was performed with cells transduced with the TKOv3 sgRNA library at an MOI of 0.4. Technical duplicates for each condition and library coverage of >400 cells per sgRNA were maintained throughout the screen procedure. 2 days post-infection, transduced cells were selected with 2 mg/ml puromycin. As above, the initial timepoint (*t*_0_) was 96h post-infection. At (*t*_6_), RP-1664 was added to the cells at 50 nM and 150 nM. Compound-containing media was refreshed every four days until the screen reached 18 days. Genomic DNA was isolated at every timepoint, and processed as described above. Sample data analysis was performed using the DrugZ (https://github.com/hart-lab/drugz)^85^ algorithm.

### Base editing screens

RPE1-hTERT *TP53*-*WT* stably expressing CBE^FNLS 44^ were transduced with lentivirus carrying a modified HBES^45^ sgRNA expression library (lacking the sensor) at MOI of ~0.3. The screen was conducted in technical triplicates, and library coverage of >1,000 cells per sgRNA was maintained at every step.

Puromycin-containing medium (2 μg/ml) was added 2 days after infection. Selection was continued until 96 h after infection, which was considered the initial time point (*t*_0_). RP-1664 was added to the cells at day 6 (*t*_6_) and 10 (*t*_10_) at the indicated concentrations and the screen was terminated at *t*_18_. Sample processing was carried out as in CRISPR/Cas9 chemogenomic screening. FASTQ files were aligned to the library reference sequences using Bowtie to generate read counts for each sample replicate at the guide level. Raw read counts from the *t*_18_ timepoint of each treatment condition were compared to DMSO control at T18 using DESeq2 to calculate log_2_FoldChange values, and the PCR2 forward primer used for each sample replicate was modeled as a covariate.

### MCF7 and CHP134 xenograft studies

MCF7 mouse xenograft studies were performed at Oncodesign under regulations from the Canadian Council on Animal Care and the National Research Council Guide. Female BALB/c Nude mice were supplemented with estradiol in drinking water (2.5 μg/mL) and gamma irradiated (1.2 Gy) 1 week and again 24–72 hours prior to inoculation in the right flank using 10 million cells. Animals were placed under blank chow for acclimatization 3 – 5 days prior to randomization. Mice were randomized into 6 groups of 6 animals each when tumor volume reached a mean of 100 – 200 mm^3^ and treatment with RP-1664 formulated chow began thereafter. Body weights and tumor volume were both measured twice a week, the latter being assessed with calipers. Tumor volume was calculated using the formula 0.52×L×W^2^, with percent tumor growth inhibition (% TGI) defined as: % TGI= ((TVvehicle_last_ – TVvehicle_day0_) - (TVtreated_last_ – TVtreated_day0_)) / (TVvehicle_last_ – TVvehicle_day0_) × 100. Percentage changes in body weight (% BW) were calculated using the formula: % BW change = (BW_last_-BW_day0_/BW_day0_) × 100.

CHP134 mouse xenograft studies were performed at Repare Therapeutics in a vivarium accredited by the Canadian Council on Animal Care with an Institutional Animal Care Committee-approved protocol. Female CB/17 SCID mice were inoculated in the right flank with 10 million cells of either parental CHP134, CHP134 *TP53*-*KO* or CHP134 *TRIM37*-*KO* and animals placed under blank chow for acclimatization 3 – 5 days prior to randomization. Mice were randomized into groups of 7 animals per cell line when tumor volume reached a mean of 100 – 150 mm^3^. Treatment with RP-1664 formulated chow began thereafter. Body weights and tumor volume were measured three times a week, the latter being assessed with digital calipers. Tumor volume, growth inhibition and body weight changes were calculated as above.

### Human neuroblastoma-derived murine xenograft studies.

The preclinical murine xenograft trials were conducted at the Children’s Hospital of Philadelphia (CHOP) and approved by the Institutional Animal Care and Use Committee (IACUC 000643). Fox Chase CB17 SCID mice (CB17/Icr-*Prkdc*^*scid*^/IcrIcoC) were purchased from Charles River Laboratories. The mice were then housed in the CHOP Department of Veterinary Research (DVR).

PDX and CDX models are maintained at CHOP and xenograft models were generated as described previously^52,72^. Briefly, viably cryopreserved neuroblastoma PDX tumor fragments or 10 million neuroblastoma cells suspended in Matrigel (Corning Catalog No. 354248) were engrafted subcutaneously into the flanks of mice and passaged once. Twelve mice per model were engrafted to ensure six mice with uniform tumor size of 200mm^3^ ± 50mm^3^ were available populate studies. Mice were allocated to either RP-1664 containing chow at 450 ppm (N=4) or standard chow (N=4) stratified by tumor volume at enrollment. One mouse per arm was sacrificed at day 14 and tumor harvested for biomarker analyses (see below). We chose a continuous dosing schedule of RP-1664 for six weeks, followed by two cycles of two weeks on and two weeks off to preliminary study the impact of intermittent dosing. Tumor volumes and weight were measure twice weekly and exposure to RP-1664 was paused if mouse weight decreased by 15% or more. Dosing was re-initiated when body weight recovered to >90%. Study endpoint was tumor volume of 2cm^3^ or day 70.

### Response evaluation and statistical methods for murine xenograft studies.

The six-week time point (end of continuous dosing) was used for all analyses. An event was defined as the quadrupling of a mouse’s tumor volume from day 0 (or baseline measurement). The exact time-to-event (in days) was estimated by interpolating between the measurements directly preceding and following the event, assuming log-linear growth. Differences in event-free survival (EFS) between experimental groups were tested using the Peto and Peto modification of the Gehan-Wilcoxon test (α = 0.05, two-sided alternative).

Initial tumor volume (V0) was measured at initiation of treatment. The mean and standard deviation of V0 was computed within each treatment group, and comparisons between treatment groups were performed using the Wilcoxon rank sum test. At subsequent tumor measurements, the relative tumor volume (RTV) was defined for each mouse as the ratio of its current tumor volume divided by V0. At the conclusion of six weeks, the minimum RTV (minRTV) for each mouse was computed across all measurements except the initial (baseline) one. The mean and standard deviation within each treatment group of minRTV was computed, and comparisons between treatments groups were performed using the Wilcoxon rank sum test.

The objective response measure (ORM) categories are progressive disease (PD, which is subdivided into progressive disease without and with growth delay, PD1 and PD2, respectively, defined only for treated mice), stable disease (SD), partial response (PR), complete response (CR), and maintained complete response (MCR).

ORM categories are defined as:

- PD when < 50% tumor regression throughout study and > 25% tumor growth at end of study
- PD1 when PD and the mouse’s time-to-event ≤ 200% the median time-to-event in control group
- PD2 when PD and the mouse’s time-to-event is > 200% the median time-to-event in control group
- SD when < 50% tumor regression throughout study and ≤ 25% tumor growth at end of study,
- PR when ≥ 50% tumor regression at any point during study, but measurable tumor throughout study period
- CR when disappearance of measurable tumor mass during the study period occurs up to two times consecutively or intermittently any number of times
- MCR when no measurable tumor mass for at least three consecutive readings at any time after treatment has been completed

Overall group response is determined by the median response among evaluable mice as follows: Each individual mouse is assigned a score from 0 to 10 based on their ORM: PD1 = 0, PD2 = 2, SD = 4, PR = 6, CR = 8, and MCR = 10, and the median for the group determines the overall response. If the median score is half-way between an ORM number category, the objective response is assigned to the lower response category (e.g., an objective response score of 9 is scored CR). Studies in which toxic deaths are greater than 25% or in which the control group is not SD or worse are considered unevaluable and are excluded from analysis. Treatment groups with PR, CR, or MCR are considered to have had an objective response. Agents inducing objective responses are considered highly active against the tested line, while agents inducing SD or PD2 are considered to have intermediate activity, and agents producing PD1 are considered to have a low level of activity against the tested line.

The average minimum relative tumor volume (minRTV) was also utilized as a tumor volume response measure. A value of 0 indicates that the tumor is no longer detectable, while values <1.0 indicate some level of tumor regression.

### Efficacy of RP-1664 in Th-MYCN immunocompetent model

Th-MYCN animal experiments were conducted at Children’s Cancer Institute and approved by the University of New South Wales Animal Care and Ethics Committee (ACEC 22/145B) according to the Animal Research Act, 1985 (New South Wales, Australia) and the Australian Code for the Care and Use of Animals for Scientific Purposes (2013).

The *Th*-*MYCN* (Tg(Th-MYCN)41Waw, 129/SvJ*Ter* backcross (ARC, Perth, Australia) mouse model of neuroblastoma overexpresses human *MYCN* in the neuroectodermal cells, and mice develop neuroblastoma as a result^53^. The mice are maintained by breeding hemizygous mice together. All experiments utilized only Th-MYCN^+/+^ mice with 8 mice per treatment group and equal numbers of both sexes. Mice were placed on control chow for acclimatization 1–5 days prior to randomization to either control, 225ppm or 400ppm RP-1664 chow once tumors reached 5mm in diameter by abdominal palpation. Body weights were measured daily and calculated based on individual body weight changes relative to the start of treatment. Treatment schedule was 14 days on, 7 days off and mice were euthanized when the tumor reached 10 mm in diameter or when signs of a thoracic tumor manifested. Blood was collected in citrate buffer (3:1) via the saphenous vein from Th-MYCN mice (N=4/arm) on day 5 at 3 timepoints (7:30am, 12:30pm and 4:30pm) for pK analysis.

### RP-1664 blood plasma level determination

Micro-sampled whole blood was collected for mouse pharmacokinetic determinations as described^86^. Samples were extracted with 4 volumes of acetonitrile containing an internal standard. The sample extracts were centrifuged, diluted 1:1 with water and quantified against a standard curve using a reversed-phase liquid chromatography gradient coupled to electrospray mass spectrometry operated in positive mode. Whole blood concentrations were converted to plasma by dividing by the mouse blood to plasma ratio of 1.03. The calculated plasma levels were converted to free plasma levels by multiplying by the fraction unbound in mouse plasma f_u_=0.125. PK parameters were calculated using non-compartmental analysis using WinNonlin version 8.5.1.3 (Certara, Pennsylvania, USA).

### p21 Immunohistochemistry

All stainings were performed at HistoWiz, Inc. (Brooklyn, NY) using the Leica Bond RX automated stainer (Leica Microsystems), following a standardized operating procedure and fully automated workflow. Samples were processed, embedded in paraffin, and sectioned at 4μm. Slides were dewaxed using xylene and alcohol-based dewaxing solutions. Epitope retrieval was performed via heat-induced epitope retrieval (HIER) using a Tris-based pH 9 solution (Leica Microsystems, AR9640) for 20 minutes at 95 °C. Tissue sections were first incubated with a peroxide block buffer (Leica Microsystems), followed by a 30-minute incubation with rabbit anti-P21 antibody (Abcam, ab109520) at a 1:200 dilution. Detection was performed using DAB rabbit secondary reagents, including polymer, DAB refine, and hematoxylin (Bond Polymer Refine Detection Kit, Leica Microsystems), according to the manufacturer’s protocol. Slides were dried, coverslipped (TissueTek-Prisma Coverslipper), and scanned using a Leica Aperio AT2 slide scanner (Leica Microsystems) at 40x magnification.

## Supplementary Material

Supplementary Files

This is a list of supplementary files associated with this preprint. Click to download.

• FortressMOAFigS2mz250307.pdf
• FortressMOAFigS1mz240403v1.pdf
• FortressMOAFigS7mz250501.pdf
• FortressMOAFigS6mz250501.pdf
• SuppVideo1CHP134DMSO.wmv
- SuppTable1ResistorCHP134.xlsx
• FortressMOAFigS3mz240820v1.pdf
• SuppTable3BaseEditRPE1.xlsx
• FortressMOAFigS4mz240808v1.pdf
• SuppVideo4RPE11664clusteringtripolar.wmv
• SuppVideo2CHP1341664.wmv
- FortressMOAFigS5mz250120v3.pdf
• SuppVideo3RPE1normaldivisiondmso.wmv
• SuppTable2SensitizerRPE1.xlsx
• SuppVideo5RPE11664exclusion.wmv
• SUUPLEMENTARY.docx

## Figures and Tables

**Table 1:** Efficacy of RP-1664 in neuroblastoma xenograft models. Details of statistical analysis used to evaluate time-to-event outcomes following treatment with RP-1664 versus control in neuroblastoma xenograft models. KM Med: Kaplan–Meier estimate of median event-free survival (EFS). EFS T – C: Absolute difference in median EFS between treatment and control groups. EFS T/C: ratio of median EFS in the treatment group to control. P-value from the Gehan–Wilcoxon test: statistical significance of differences in EFS. minRTV: Average minimum relative tumor volume observed in each group ±SD. Med resp: Objective response measures. CR = complete response, MCR = maintained CR, and PD/PD1/PD2 = progressive disease. See Methods for more information.

Model	Grp	N	KM med	EFS T-C	EFS T/C	*P*-value Gehan-Wilcoxon	minRTV mean ±SD	minRTV *P*-value	Med resp
COG-N-415X	Vehicle	3	8.61				2.84+/−0.88		PD
	RP-1664	3	20.62	12.01	2.39	0.0339	1.68+/−0.24	0.0809	PD2

COG-N-421X	Vehicle	3	18.52				1.4+/−0.26		PD
	RP-1664	3	104.32	85.8	5.63	0.0339	0+/−0	0.0636	MCR

COG-N-424X	Vehicle	3	8.38				1.89+/−0.5		PD
	RP-1664	3	>84	75.62	10.02	0.0339	0+/−0	0.0636	MCR

COG-N-453X	Vehicle	3	7.85				1.92+/−0.35		PD
	RP-1664	3	44.65	36.8	5.69	0.031	0.63+/−0.34	0.0809	PD2

COG-N-519X	Vehicle	3	14.23				1.53+/−0.2		PD
	RP-1664	3	88.66	74.43	6.23	0.0339	0+/−0	0.0636	CR

COG-N-557X	Vehicle	3	26.6				1.11+/−0.34		PD
	RP-1664	3	74.45	47.85	2.8	0.1175	0.82+/−0.55	0.6625	PD2

COG-N-561X	Vehicle	3	12.98				1.76+/−0.27		PD
	RP-1664	3	80.88	67.9	6.23	0.0339	0.06+/−0.1	0.0765	CR

COG-N-619X	Vehicle	3	22.62				1.43+/−0.07		PD
	RP-1664	3	45.49	22.87	2.01	0.0339	0.9+/−0.46	0.1904	PD2

FELIX PDX	Vehicle	3	13.17				1.54+/−0.68		PD
	RP-1664	3	77.29	64.12	5.87	0.0339	0+/−0	0.0636	CR

NB-EBC1	Vehicle	3	4.54				2.91+/−0.85		PD
	RP-1664	3	80.11	75.57	17.65	0.0339	0.29+/−0.5	0.0765	CR

6FN	Vehicle	3	14.32				1.39+/−0.19		PD
	RP-1664	3	22.36	8.04	1.56	0.0339	1.1+/−0.13	0.0809	PD1

NB-SD	Vehicle	3	12.64				1.49+/−0.05		PD
	RP-1664	3	91.73	79.09	7.26	0.0339	0.32+/−0.21	0.0809	PR

NB-1643	Vehicle	3	6.13				2.47+/−0.77		PD
	RP-1664	3	96.43	90.3	15.73	0.0339	0.06+/−0.1	0.0765	CR

NB-1691	Vehicle	3	8.3				1.52+/−0.43		PD
	RP-1664	3	79.77	71.47	9.61	0.5127	1.44+/−1.08	1	PD2

SK-N-AS	Vehicle	3	13.74				1.5+/−0.36		PD
	RP-1664	3	64.85	51.11	4.72	0.0339	1.3+/−0.24	0.6625	PD2

## References

[1] Matthay K. K. Neuroblastoma. Nat Rev Dis Primers 2, 16078 (2016). 27830764 10.1038/nrdp.2016.78

[2] Bown N. Gain of chromosome arm 17q and adverse outcome in patients with neuroblastoma. N Engl J Med 340, 1954–1961 (1999). 10379019 10.1056/NEJM199906243402504

[3] Gilbert F. Human neuroblastomas and abnormalities of chromosomes 1 and 17. Cancer Res 44, 5444–5449 (1984). 6488196

[4] Morowitz M. Detection of single-copy chromosome 17q gain in human neuroblastomas using real-time quantitative polymerase chain reaction. Mod Pathol 16, 1248–1256 (2003). 14681326 10.1097/01.MP.0000097364.64566.81

[5] Mlakar V. 17q Gain in Neuroblastoma: A Review of Clinical and Biological Implications. Cancers (Basel) 16, 338 (2024). 38254827 10.3390/cancers16020338 PMC10814316

[6] Pugh T. J. The genetic landscape of high-risk neuroblastoma. Nat Genet 45, 279–284 (2013). 23334666 10.1038/ng.2529 PMC3682833

[7] Ho N. Delineation of the frequency and boundary of chromosomal copy number variations in paediatric neuroblastoma. Cell Cycle 17, 749–758 (2018). 29353549 10.1080/15384101.2017.1421875 PMC5969554

[8] Mosse Y. P. High-resolution detection and mapping of genomic DNA alterations in neuroblastoma. Genes Chromosomes Cancer 43, 390–403 (2005). 15892104 10.1002/gcc.20198

[9] Yeow Z. Y. Targeting TRIM37-driven centrosome dysfunction in 17q23-amplified breast cancer. Nature 585, 447–452 (2020). 32908313 10.1038/s41586-020-2690-1 PMC7597367

[10] Meitinger F. TRIM37 controls cancer-specific vulnerability to PLK4 inhibition. Nature 585, 440–446 (2020). 32908304 10.1038/s41586-020-2710-1 PMC7501188

[11] Breslow D. K. & Holland A. J. Mechanism and Regulation of Centriole and Cilium Biogenesis. Annu Rev Biochem 88, 691–724 (2019). 30601682 10.1146/annurev-biochem-013118-111153 PMC6588485

[12] Nigg E. A. & Holland A. J. Once and only once: mechanisms of centriole duplication and their deregulation in disease. Nat Rev Mol Cell Biol 19, 297–312 (2018). 29363672 10.1038/nrm.2017.127 PMC5969912

[13] Habedanck R., Stierhof Y.-D., Wilkinson C. J. & Nigg E. A. The Polo kinase Plk4 functions in centriole duplication. Nat Cell Biol 7, 1140–1146 (2005). 16244668 10.1038/ncb1320

[14] Bettencourt-Dias M. SAK/PLK4 is required for centriole duplication and flagella development. Curr Biol 15, 2199–2207 (2005). 16326102 10.1016/j.cub.2005.11.042

[15] Moyer T. C. & Holland A. J. PLK4 promotes centriole duplication by phosphorylating STIL to link the procentriole cartwheel to the microtubule wall. Elife 8, e46054 (2019). 31115335 10.7554/eLife.46054 PMC6570480

[16] Holland A. J. Polo-like kinase 4 controls centriole duplication but does not directly regulate cytokinesis. Mol Biol Cell 23, 1838–1845 (2012). 22456511 10.1091/mbc.E11-12-1043 PMC3350549

[17] Lee M., Seo M. Y., Chang J., Hwang D. S. & Rhee K. PLK4 phosphorylation of CP110 is required for efficient centriole assembly. Cell Cycle 16, 1225–1234 (2017). 28562169 10.1080/15384101.2017.1325555 PMC5499911

[18] Holland A. J. The autoregulated instability of Polo-like kinase 4 limits centrosome duplication to once per cell cycle. Genes Dev 26, 2684–2689 (2012). 23249732 10.1101/gad.207027.112 PMC3533073

[19] Holland A. J., Lan W., Niessen S., Hoover H. & Cleveland D. W. Polo-like kinase 4 kinase activity limits centrosome overduplication by autoregulating its own stability. J Cell Biol 188, 191–198 (2010). 20100909 10.1083/jcb.200911102 PMC2813471

[20] Rogers G. C., Rusan N. M., Roberts D. M., Peifer M. & Rogers S. L. The SCF Slimb ubiquitin ligase regulates Plk4/Sak levels to block centriole reduplication. J Cell Biol 184, 225–239 (2009). 19171756 10.1083/jcb.200808049 PMC2654306

[21] Klebba J. E. Polo-like kinase 4 autodestructs by generating its Slimb-binding phosphodegron. Curr Biol 23, 2255–2261 (2013). 24184097 10.1016/j.cub.2013.09.019 PMC3844517

[22] Domínguez-Calvo A., Gönczy P., Holland A. J. & Balestra F. R. TRIM37: a critical orchestrator of centrosome function. Cell Cycle 20, 2443–2451 (2021). 34672905 10.1080/15384101.2021.1988289 PMC8794516

[23] Meitinger F. Control of cell proliferation by memories of mitosis. Science 383, 1441–1448 (2024). 38547292 10.1126/science.add9528 PMC11621110

[24] Cuella-Martin R. 53BP1 Integrates DNA Repair and p53-Dependent Cell Fate Decisions via Distinct Mechanisms. Mol Cell 64, 51–64 (2016). 27546791 10.1016/j.molcel.2016.08.002 PMC5065530

[25] Meitinger F. 53BP1 and USP28 mediate p53 activation and G1 arrest after centrosome loss or extended mitotic duration. J Cell Biol 214, 155–166 (2016). 27432897 10.1083/jcb.201604081 PMC4949453

[26] Lambrus B. G. A USP28–53BP1-p53-p21 signaling axis arrests growth after centrosome loss or prolonged mitosis. J Cell Biol 214, 143–153 (2016). 27432896 10.1083/jcb.201604054 PMC4949452

[27] Vallée F. Discovery of RP-1664: A First-in-Class Orally Bioavailable, Selective PLK4 Inhibitor. J Med Chem (2025) doi:10.1021/acs.jmedchem.5c00529. 40378279

[28] Tkach J. M. Global cellular response to chemical perturbation of PLK4 activity and abnormal centrosome number. Elife 11, e73944 (2022). 35758262 10.7554/eLife.73944 PMC9236612

[29] Mason J. M. Functional characterization of CFI-400945, a Polo-like kinase 4 inhibitor, as a potential anticancer agent. Cancer Cell 26, 163–176 (2014). 25043604 10.1016/j.ccr.2014.05.006

[30] Moyer T. C., Clutario K. M., Lambrus B. G., Daggubati V. & Holland A. J. Binding of STIL to Plk4 activates kinase activity to promote centriole assembly. J Cell Biol 209, 863–878 (2015). 26101219 10.1083/jcb.201502088 PMC4477857

[31] Lei Q. YLT-11, a novel PLK4 inhibitor, inhibits human breast cancer growth via inducing maladjusted centriole duplication and mitotic defect. Cell Death Dis 9, 1066 (2018). 30337519 10.1038/s41419-018-1071-2 PMC6194023

[32] Holland A. J. & Cleveland D. W. Polo-like kinase 4 inhibition: a strategy for cancer therapy? Cancer Cell 26, 151–153 (2014). 25117704 10.1016/j.ccr.2014.07.017 PMC4148003

[33] Guderian G., Westendorf J., Uldschmid A. & Nigg E. A. Plk4 trans-autophosphorylation regulates centriole number by controlling betaTrCP-mediated degradation. J Cell Sci 123, 2163–2169 (2010). 20516151 10.1242/jcs.068502

[34] Noordermeer S. M. The shieldin complex mediates 53BP1-dependent DNA repair. Nature 560, 117–121 (2018). 30022168 10.1038/s41586-018-0340-7 PMC6141009

[35] Joshi H. C., Palacios M. J., McNamara L. & Cleveland D. W. Gamma-tubulin is a centrosomal protein required for cell cycle-dependent microtubule nucleation. Nature 356, 80–83 (1992). 1538786 10.1038/356080a0

[36] Paulson J. R. & Taylor S. S. Phosphorylation of histones 1 and 3 and nonhistone high mobility group 14 by an endogenous kinase in HeLa metaphase chromosomes. J Biol Chem 257, 6064–6072 (1982). 6281254

[37] Wong Y. L. Cell biology. Reversible centriole depletion with an inhibitor of Polo-like kinase 4. Science 348, 1155–1160 (2015). 25931445 10.1126/science.aaa5111 PMC4764081

[38] Surrey L. F. Clinical utility of custom-designed NGS panel testing in pediatric tumors. Genome Med 11, 32 (2019). 31133068 10.1186/s13073-019-0644-8 PMC6537185

[39] Hart T. Evaluation and Design of Genome-Wide CRISPR/SpCas9 Knockout Screens. G3 (Bethesda) 7, 2719–2727 (2017). 28655737 10.1534/g3.117.041277 PMC5555476

[40] Olivieri M. & Durocher D. Genome-scale chemogenomic CRISPR screens in human cells using the TKOv3 library. STAR Protoc 2, 100321 (2021). 33598657 10.1016/j.xpro.2021.100321 PMC7868615

[41] Tinel A. & Tschopp J. The PIDDosome, a protein complex implicated in activation of caspase-2 in response to genotoxic stress. Science 304, 843–846 (2004). 15073321 10.1126/science.1095432

[42] Fava L. L. The PIDDosome activates p53 in response to supernumerary centrosomes. Genes Dev 31, 34–45 (2017). 28130345 10.1101/gad.289728.116 PMC5287111

[43] Gao Z., Shao Y. & Jiang X. Essential roles of the Bcl-2 family of proteins in caspase-2-induced apoptosis. J Biol Chem 280, 38271–38275 (2005). 16172118 10.1074/jbc.M506488200

[44] Zafra M. P. Optimized base editors enable efficient editing in cells, organoids and mice. Nat Biotechnol 36, 888–893 (2018). 29969439 10.1038/nbt.4194 PMC6130889

[45] Sánchez-Rivera F. J. Base editing sensor libraries for high-throughput engineering and functional analysis of cancer-associated single nucleotide variants. Nat Biotechnol 40, 862–873 (2022). 35165384 10.1038/s41587-021-01172-3 PMC9232935

[46] Zhang C. Identification of KIFC1 as a putative vulnerability in lung cancers with centrosome amplification. Cancer Gene Ther 31, 1559–1570 (2024). 39179685 10.1038/s41417-024-00824-1 PMC11489082

[47] Kwon M. Mechanisms to suppress multipolar divisions in cancer cells with extra centrosomes. Genes Dev 22, 2189–2203 (2008). 18662975 10.1101/gad.1700908 PMC2518815

[48] Godinho S. A. & Pellman D. Causes and consequences of centrosome abnormalities in cancer. Philos Trans R Soc Lond B Biol Sci 369, 20130467 (2014). 25047621 10.1098/rstb.2013.0467 PMC4113111

[49] Basto R. Centrosome amplification can initiate tumorigenesis in flies. Cell 133, 1032–1042 (2008). 18555779 10.1016/j.cell.2008.05.039 PMC2653712

[50] Sabino D. Moesin is a major regulator of centrosome behavior in epithelial cells with extra centrosomes. Curr Biol 25, 879–889 (2015). 25772448 10.1016/j.cub.2015.01.066 PMC4386030

[51] Kendsersky N. M. The B7-H3–Targeting Antibody–Drug Conjugate m276-SL-PBD Is Potently Effective Against Pediatric Cancer Preclinical Solid Tumor Models. Clinical Cancer Research 27, 2938–2946 (2021). 33619171 10.1158/1078-0432.CCR-20-4221 PMC8127361

[52] Houghton P. J. The pediatric preclinical testing program: description of models and early testing results. Pediatr Blood Cancer 49, 928–940 (2007). 17066459 10.1002/pbc.21078

[53] Weiss W. A., Aldape K., Mohapatra G., Feuerstein B. G. & Bishop J. M. Targeted expression of MYCN causes neuroblastoma in transgenic mice. EMBO J 16, 2985–2995 (1997). 9214616 10.1093/emboj/16.11.2985 PMC1169917

[54] Hansford L. M. Mechanisms of embryonal tumor initiation: distinct roles for MycN expression and MYCN amplification. Proc Natl Acad Sci U S A 101, 12664–12669 (2004). 15314226 10.1073/pnas.0401083101 PMC515113

[55] Burkhart C. A. Effects of MYCN antisense oligonucleotide administration on tumorigenesis in a murine model of neuroblastoma. J Natl Cancer Inst 95, 1394–1403 (2003). 13130115 10.1093/jnci/djg045

[56] Bourmoum M. β-catenin mediates growth defects induced by centrosome loss in a subset of APC mutant colorectal cancer independently of p53. PLoS One 19, e0295030 (2024). 38324534 10.1371/journal.pone.0295030 PMC10849215

[57] Fielding A. B., Lim S., Montgomery K., Dobreva I. & Dedhar S. A critical role of integrin-linked kinase, ch-TOG and TACC3 in centrosome clustering in cancer cells. Oncogene 30, 521–534 (2011). 20838383 10.1038/onc.2010.431

[58] Yang Z., Loncarek J., Khodjakov A. & Rieder C. L. Extra centrosomes and/or chromosomes prolong mitosis in human cells. Nat Cell Biol 10, 748–751 (2008). 18469805 10.1038/ncb1738 PMC2430725

[59] Drosopoulos K., Tang C., Chao W. C. H. & Linardopoulos S. APC/C is an essential regulator of centrosome clustering. Nat Commun 5, 3686 (2014). 24751481 10.1038/ncomms4686

[60] Leber B. Proteins required for centrosome clustering in cancer cells. Sci Transl Med 2, 33ra38 (2010). 10.1126/scitranslmed.3000915 20505215

[61] Rhys A. D. Loss of E-cadherin provides tolerance to centrosome amplification in epithelial cancer cells. J Cell Biol 217, 195–209 (2018). 29133484 10.1083/jcb.201704102 PMC5748979

[62] Schnepp R. W. A LIN28B-RAN-AURKA Signaling Network Promotes Neuroblastoma Tumorigenesis. Cancer Cell 28, 599–609 (2015). 26481147 10.1016/j.ccell.2015.09.012 PMC4643330

[63] Powers J. T. Multiple mechanisms disrupt the let-7 microRNA family in neuroblastoma. Nature 535, 246–251 (2016). 27383785 10.1038/nature18632 PMC4947006

[64] Attiyeh E. F. Chromosome 1p and 11q Deletions and Outcome in Neuroblastoma. N Engl J Med 353, 2243–2253 (2005). 16306521 10.1056/NEJMoa052399

[65] Nikonova A. S., Astsaturov I., Serebriiskii I. G., Dunbrack R. L. & Golemis E. A. Aurora A kinase (AURKA) in normal and pathological cell division. Cell Mol Life Sci 70, 661–687 (2013). 22864622 10.1007/s00018-012-1073-7 PMC3607959

[66] Slack A. D., Chen Z., Ludwig A. D., Hicks J. & Shohet J. M. MYCN-directed centrosome amplification requires MDM2-mediated suppression of p53 activity in neuroblastoma cells. Cancer Res 67, 2448–2455 (2007). 17363562 10.1158/0008-5472.CAN-06-1661

[67] Sugihara E. Enhanced expression of MYCN leads to centrosome hyperamplification after DNA damage in neuroblastoma cells. Oncogene 23, 1005–1009 (2004). 14647433 10.1038/sj.onc.1207216

[68] Fukushi D. Centrosome amplification is correlated with ploidy divergence, but not with MYCN amplification, in neuroblastoma tumors. Cancer Genet Cytogenet 188, 32–41 (2009). 19061778 10.1016/j.cancergencyto.2008.08.014

[69] Marín N. M. High prevalence and dependence of centrosome clustering in mesenchymal tumors and leukemia. Preprint at 10.1101/2023.03.13.532472 (2023).

[70] Evans L. T. ANKRD26 recruits PIDD1 to centriolar distal appendages to activate the PIDDosome following centrosome amplification. EMBO J 40, e105106 (2021). 33350495 10.15252/embj.2020105106 PMC7883295

[71] Guo Y., Srinivasula S. M., Druilhe A., Fernandes-Alnemri T. & Alnemri E. S. Caspase-2 induces apoptosis by releasing proapoptotic proteins from mitochondria. J Biol Chem 277, 13430–13437 (2002). 11832478 10.1074/jbc.M108029200

[72] Smith M. A. Lessons learned from 20 years of preclinical testing in pediatric cancers. Pharmacology & Therapeutics 264, 108742 (2024). 39510293 10.1016/j.pharmthera.2024.108742 PMC12085791

[73] Infarinato N. R. The ALK/ROS1 Inhibitor PF-06463922 Overcomes Primary Resistance to Crizotinib in ALK-Driven Neuroblastoma. Cancer Discovery 6, 96–107 (2016). 26554404 10.1158/2159-8290.CD-15-1056 PMC4707106

[74] Goldsmith K. C. Lorlatinib with or without chemotherapy in ALK-driven refractory/relapsed neuroblastoma: phase 1 trial results. Nat Med 29, 1092–1102 (2023). 37012551 10.1038/s41591-023-02297-5 PMC10202811

[75] Zimmermann M. CRISPR screens identify genomic ribonucleotides as a source of PARP-trapping lesions. Nature 559, 285–289 (2018). 29973717 10.1038/s41586-018-0291-z PMC6071917

[76] Egolf L. E. Germline 16p11.2 Microdeletion Predisposes to Neuroblastoma. The American Journal of Human Genetics 105, 658–668 (2019). 31474320 10.1016/j.ajhg.2019.07.020 PMC6731370

[77] Lee S. NGSCheckMate: software for validating sample identity in next-generation sequencing studies within and across data types. Nucleic Acids Res 45, e103 (2017). 28369524 10.1093/nar/gkx193 PMC5499645

[78] Boeva V. Control-FREEC: a tool for assessing copy number and allelic content using next-generation sequencing data. Bioinformatics 28, 423–425 (2012). 22155870 10.1093/bioinformatics/btr670 PMC3268243

[79] Talevich E., Shain A. H., Botton T. & Bastian B. C. CNVkit: Genome-Wide Copy Number Detection and Visualization from Targeted DNA Sequencing. PLoS Comput Biol 12, e1004873 (2016). 27100738 10.1371/journal.pcbi.1004873 PMC4839673

[80] McKenna A. The Genome Analysis Toolkit: a MapReduce framework for analyzing next-generation DNA sequencing data. Genome Res 20, 1297–1303 (2010). 20644199 10.1101/gr.107524.110 PMC2928508

[81] Shapiro J. A. OpenPBTA: The Open Pediatric Brain Tumor Atlas. Cell Genom 3, 100340 (2023). 37492101 10.1016/j.xgen.2023.100340 PMC10363844

[82] Dobin A. STAR: ultrafast universal RNA-seq aligner. Bioinformatics 29, 15–21 (2013). 23104886 10.1093/bioinformatics/bts635 PMC3530905

[83] Li B. & Dewey C. N. RSEM: accurate transcript quantification from RNA-Seq data with or without a reference genome. BMC Bioinformatics 12, 323 (2011). 21816040 10.1186/1471-2105-12-323 PMC3163565

[84] Conant D. Inference of CRISPR Edits from Sanger Trace Data. CRISPR J 5, 123–130 (2022). 35119294 10.1089/crispr.2021.0113

[85] Colic M. Identifying chemogenetic interactions from CRISPR screens with drugZ. Genome Med 11, 52 (2019). 31439014 10.1186/s13073-019-0665-3 PMC6706933

[86] Bateman K. P. Reduction of animal usage by serial bleeding of mice for pharmacokinetic studies: application of robotic sample preparation and fast liquid chromatography-mass spectrometry. J Chromatogr B Biomed Sci Appl 754, 245–251 (2001). 11318421 10.1016/s0378-4347(00)00612-5

